# *TERT* Amplification a Risk Stratification Marker in Papillary Thyroid Carcinoma, Significantly Correlated with Tumor Recurrence and Survival

**DOI:** 10.1007/s12022-025-09853-4

**Published:** 2025-04-24

**Authors:** Sara Gil-Bernabé, Noa Feás-Rodríguez, Enrique Pérez-Riesgo, Miriam Corraliza-Gómez, Joaquín Fra Rodríguez, Ginesa García-Rostán

**Affiliations:** 1 Institute of Biomedicine and Molecular Genetics (IBGM), Valladolid University, Valladolid, Spain; 2 School of Medicine, Valladolid University, Valladolid, Spain; 3 European Miguel de Cervantes University, Valladolid, Spain; 4 University of Cádiz, Cádiz, Spain; 5 Hospital Río Hortega, Valladolid, Spain

**Keywords:** *TERT* amplification, Thyroid cancer, Metastases, Prognosis, Tumor recurrence, Survival

## Abstract

Few studies have analyzed the prevalence of *TERT* amplification in thyroid cancer, showing discrepancies in various topics. The impact on tumor recurrence and patient survival in papillary thyroid carcinoma (PTC) remains unknown. Thirteen cancer cell lines and 215 tumor samples from 91 patients, who underwent surgery for PTC (41), poorly differentiated thyroid carcinoma (PDC = 15), or anaplastic thyroid carcinoma (ATC = 35), were analyzed. Clonality, spread with tumor dedifferentiation or metastatic PTC cells, and coexistence with *TERTp*, *BRAF*, *RAS*, and *PIK3CA* mutations were also investigated. *TERT* amplification was found in 17%, 20%, and 17% of the PTC, PDC, and ATC, respectively. It was more frequent in follicular variant PTC and PTC with distant metastases (86%, *P* = 0.0448). The cell lines HTh74, SW1736, and T242 had amplification. In PTC, *TERT* amplification was a subclonal event. The increase in *TERT* copy number spread in all cases with metastatic PTC cells. In 67% of the PDC and 100% of the ATC, *TERT* activation segregated with tumor dedifferentiation. *TERT* amplification correlated with *TERTp* mutations in PTC (*P* = 0.0313) and *PIK3CA* mutations in ATC (*P* = 0.0272). *TERT* amplification significantly correlated with vascular invasion (*P* = 0.03637), distant metastases at diagnosis and/or follow-up (*P* = 0.04482), metachronous distant metastases (*P* = 0.03131), death patient status (*P* = 0.000829), stage at diagnosis (*P* = 0.01995), and stage III/IV at last follow-up (*P* = 0.01552). *TERT* amplification associated independently with tumor-related recurrence and death. Our study shows that PTC can be stratified into clinically prognostic relevant categories based on the presence or not of *TERT* amplification in the cells.

**Supplementary Information:**

The online version contains supplementary material available at 10.1007/s12022-025-09853-4.

## Introduction

During cancer progression and dedifferentiation, several genetic changes including mutations, amplifications, and deletions can occur within the cancer genome. While much attention has focused on the identification of mutations, alterations of DNA copy number or copy number variations (CNV) within the genome of cancer cells have been the subject of fewer studies. CNV, gains or losses of chromosomal regions, including whole chromosomal arms, are, however, quite frequent in many cancers and have the potential to deeply affect gene expression patterns and regulatory pathways. Cancer cells with amplification of genes encoding oncogenic products show selective growth advantages, leading to increased oncogene expression [1–4].

*TERT* is located in the short arm of chromosome 5, which is one of the most frequent arm level regions amplified in solid tumors (13.2%) and cancer cell lines [1–4]. In the case of thyroid cancer, the cancer genome atlas (TCGA) research network has studied nearly 500 papillary thyroid carcinomas (PTCs) unveiling 18 significant somatic arm level CNV, including somatic copy number gains or amplifications at chromosome 5p in 4.4% (22/495) of the cases [5]. Even though understanding of *TERT* regulation and telomerase reactivation in thyroid cancer is crucial to decipher its role in tumor pathogenesis, progression, and aggressiveness, since 2014 that the TCGA research network approached the genomic characterization of PTCs, very few original research studies have focused on investigating somatic *TERT* CNV (copy number gains or amplification) as a mechanism of telomerase reactivation in thyroid follicular cell tumorigenesis [6–10]. The reported overall prevalence of somatic *TERT* copy number gains or amplification in PTCs ranges between 2 and 29.4%. The wide prevalence range observed is likely due to the design of the studies published so far, particularly with regard to the number of total cases and of each tumor histotype analyzed. Prevalence variability might also be due to the lack of discrimination in some studies between lymph node metastases (LNMs) and distant metastases (DMs) that were included, in different proportions, in the same group or inclusively all together with primary tumors (pTs), even though it is known that they do not have the same prognostic impact. The studies published on PTCs also differ as to the higher prevalence of somatic *TERT* CNV in particular variants of PTC (follicular variant - FV-PTC), the concurrent or mutually exclusive nature of *TERT* promoter mutations (*TPM*) and *TERT* amplification, and the greater or lesser frequency of somatic *TERT* copy number gains or amplification in *BRAF*-mutated or *RAS*-mutated tumors [5–9]. Despite the discrepancies observed in the limited number of studies that have included PTCs and explored alternative mechanisms to *TPM* in relation to TERT expression, there is a consensus that genetic alterations related to TERT re-expression and telomerase activation are late events in thyroid tumorigenesis. These alterations tend to accumulate in advanced, high-stage, and clinically aggressive thyroid cancers [5–9]. In case series or subset of cases in which advanced, high stage, clinically aggressive thyroid cancers are overrepresented either as pTs or as DMs [9], the prevalence of *TERT* amplification results much higher (29.4%) than in series in which the commonest histotype, as occurs in the TCGA thyroid research project [5], is a conventional, low/intermediate risk PTC (4.4–5%).

None of the published studies has analyzed advanced stage, radioiodine-resistant PTCs with matched synchronous and/or metachronous DMs, which precludes the possibility of inferring tumor evolution. Likewise, none of the reported studies, involving PTCs, has investigated the relationship between *TERT* amplification or *TERT* amplification + *TPM* and clinical-pathological parameters of poor outcome in thyroid patients. Moreover, the impact of *TERT* amplification alone or in combination with *TPM* on tumor relapse and patient survival has not been assessed in PTCs. Only Paulsson et al. have approached a detailed in-depth study of the *TERT* aberrancies (*TPM*, *TERT* CNV, and *TERT* methylation) underlying *TERT* upregulation in follicular thyroid carcinomas (FTC) demonstrating that *TERT* copy number gains, *TERT* methylation, and *TERT* expression are independently associated with FTC-related relapse. Based on their results, Paulsson et al. suggest that the finding of *TERT* expression joined with *TERT* aberrancies in postoperative genetic analyses of tumor material could help to pinpoint cases with a putative malignant molecular phenotype, even in the absence of histopathological malignancy [6].

In an attempt to shed some light on the discrepancies reported so far with respect to *TERT* amplification, confirm its consideration as a late event in thyroid carcinogenesis, associated with aggressive, advanced stage carcinomas, and simultaneously define its impact on the outcome and survival of PTC patients, in this study, we analyzed 215 tumor samples from 91 patients, who underwent surgery for PTC, poorly differentiated thyroid carcinoma (PDC), or anaplastic thyroid carcinoma (ATC). To assess putative differences in the prevalence and clinical effect of *TERT* amplification depending on the stage of the tumor genotyped, the PTCs were subdivided into 2 subgroups with similar number of patients, enough representative for statistical analysis. A group of PTCs with paired LNMs, which did not develop DMs at diagnosis and/or during the follow-up, and a group of PTCs with matched synchronous and/or metachronous DMs. To verify the concurrent or mutually exclusive nature of *TERT* amplification and *TPM* and the alleged association of *TERT* amplification with other oncogenic drivers in thyroid carcinogenesis, we investigated in the same cohort of tumors the presence of *TPM*, *BRAF*, *HRAS*, *KRAS*, *NRAS*, and *PIK3CA* mutations. For comparison with PTCs, PDCs, and ATCs, 13 thyroid cancer cell lines, derived from pTs with different degrees of differentiation or originated in DMs or LNMs of thyroid tumors, were evaluated for *TERT* amplification. Whenever feasible, the clonality and evolutionary trajectory or spread of each of the oncogenic drivers investigated were evaluated. The degree of molecular heterogeneity present in all 3 histotypes (PTC, PDC, and ATC) was also measured.

## Material and Methods

All the methodological approaches included in this study were in agreement with international and institutional ethical standards. Processing of samples and of patient information proceeded in accordance with institutional review-board approved protocols.

### Study Population. Clinical-Pathological Parameters

A total of 215 formalin-fixed paraffin-embedded (FFPE) tumor samples from 91 patients, who underwent surgery for PTC (*n* = 41), PDC (*n* = 15), or ATC (*n* = 35) were analyzed. Tumors were retrieved from the files of 7 different Pathology Departments of Spanish University Hospitals. All histologic diagnoses were reviewed according to established histologic criteria [11–13] by G. G-R. PTCs were divided into two groups. PTCs with available tissue for genotyping from paired LNMs, which did not develop blood borne DMs at diagnosis and/or during the follow-up (21 PTCs without DMs). PTCs with available tissue for genotyping from matched synchronous and/or metachronous DMs (20 PTCs with DMs). The immunoreactivity for thyroid transcription factor 1 and/or thyroglobulin confirmed the thyroid origin of the DMs. Staining for cytokeratin 19 was used as a marker of papillary differentiation. In total, 22 CL-PTCs and 19 FV-PTCs were characterized. In the latter group, 17 were infiltrative FV-PTCs and 2 were encapsulated invasive PTCs. To decode the pattern of intratumoral molecular heterogeneity (ITGH) and the clonality in the activation of oncogenic drivers, whenever the availability of tissue for genotyping was feasible, a comprehensive multiregional, geographical genotyping of pTs, LNMs, and DMs was approached. More than one area of pT was genotyped in 41% of the PTCs. Focal changes in the predominant pattern of growth in the pT were characterized in 26% of the PTCs. More than one LNM and more than one synchronous and/or metachronous DM were investigated in 36% and 30% of the PTCs, respectively. More than one area of pT was analyzed in 40% of the PDCs and 20% of the ATCs. A concurrent better differentiated thyroid tumor histotype within the pT was characterized in 33% of the PDCs and 17% of the ATCs. Detailed clinical and follow-up information was available in the 41 PTCs (see Table 1). The 15 PDCs and 35 ATCs included in the study represent a subgroup of aggressive thyroid cancers integrated within an ongoing project in PDCs and ATCs, and, thus, the process of clinical data collection is not completed and the follow-up of PDCs has not been closed yet. Patients were staged following the recommendations of the American Joint Committee on Cancer (AJCC) 8th edition [14] and managed according to standard clinical protocols. No prior radiation exposure was documented in any of the patients. Likewise, none of the patients received targeted therapy before genetic analyses.

**Table 1:** Pathological and clinical features of the 41 papillary thyroid carcinomas analyzed in this study

Clinical-pathological features	CL-PTC (*n* = 22)	FV-PTC^$^ (*n* = 19)	Total (*n* = 41)
Foci of infiltrative insulae of tumor cells at the advancing edge of the tumor	11 (50)	10 (59)^#^	21 (54)^**#**^
Focal tall cell appearance	8 (36)	6 (32)	14 (34)
Age ≥ 45	12 (55)	10 (53)	22 (54)
Age ≥ 55	8 (36)	7 (37)	15 (37)
Male sex	7 (32)	7 (37)	14 (34)
Multifocality	11 (50)	13 (76)^#^	24 (62)^**#**^
Multifocality + tumor size ≥ 5 cm	16 (73)	14 (82)^#^	30 (77)^#^
Vascular invasion	5 (23)	14 (74)	19 (46)
Extrathyroidal extension	10 (45)	9 (53)^#^	19 (49)^**#**^
Lymph node metastases	22 (100)	10 (53)	32 (78)
Lymph node metastases at diagnosis	21 (95)	8 (42)	29 (71)
Lymph node metastases at follow-up	9 (41)	3 (16)	12 (29)
Distant metastases	7 (32)	13 (68)	20 (49)
Distant metastases at diagnosis	1 (5)	8 (42)	9 (22)
Distant metastases at follow-up	7 (32)	11 (58)	18 (44)
Recurrence	11 (50)	12 (63)	23 (56)
Patient status-DOD	5 (23)	10 (53)	15 (37)
Stage at diagnosis AJCC 8th ed			
Stage I	14 (64)	10 (53)	24 (59)
Stage II	6 (27)	1 (5)	7 (17)
Stage III	2 (9)	0	2 (5)
Stage IV	0	8 (42)	8 (20)
Stage III/IV at last follow-up AJCC 8th ed	3 (14)	9 (47)	12 (29)
Stage at last follow-up AJCC 8th ed			
Stage I	9 (41)	6 (32)	15 (37)
Stage II	10 (45)	4 (21)	14 (34)
Stage III	1 (5)	0	1 (2)
Stage IV	2 (9)	9 (47)	11 (27)
Mean follow-up (months/years)	149/12	151/13	150 /13
Patient status - (alive/dead)	17/5	9/10	26/15
NED	12 (55)	7 (37)	19 (46)
AWD	5 (23)	2 (11)	7 (17)
DOD	5 (23)	10 (53)	15 (37)

The numbers in parentheses indicate a percentage value

Abbreviations: *AJCC* American Joint Committee on Cancer, *NED* no evidence of disease, *AWD* alive with disease, *DOD* death of disease

^$^17 FV-PTCs were infiltrative FV-PTCs and 2 were encapsulated invasive FV-PTCs

^**#**^No available information in two cases

For comparison with PTCs, PDCs, and ATCs, 13 thyroid cancer cell lines, derived from pTs with different degrees of differentiation or originated in DMs or LNMs of thyroid tumors, were evaluated for *TERT* amplification and mutations at *TERT*, *BRAF*, *RAS*, and *PIK3CA*. The study included 7 cell lines derived from ATC pTs: HTh74, HTh83, 8505C, C643, CAL-62, SW1736, and TCO-1; 1 cell line derived from a LNM of ATC: BHT-101; 2 cell lines derived from pTs of PDCs: T243 and B-CPAP; 1 cell line derived from a pT of PTC: TPC-1; 1 cell line derived from a LNM of PTC: MDA-T41; 1 cell line derived from a pleural DM of PTC: CUTC5.

### Methods

See “online resource 1” for detailed information on the different methods applied in the study.

## Results

### *TERT* Amplification and/or *TPM*

**Fig. 1:**
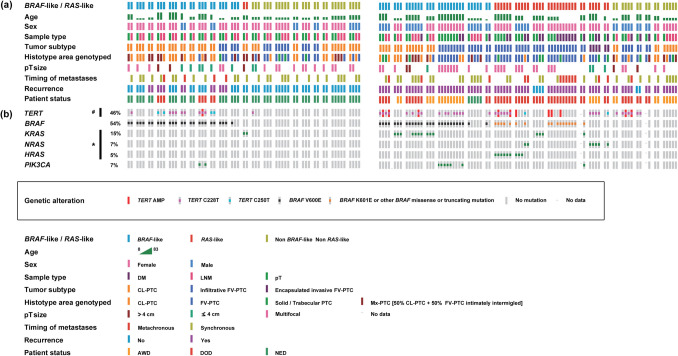
Pattern of oncogenic activation in 21 PTCs with paired LNMs that did not develop DMs at diagnosis and/or during the follow-up (left hand side of the figure) and 20 PTCs with matching synchronous and/or metachronous DMs (right hand side of the figure). To facilitate the comprehension of the clonality or subclonality in the activation of the different oncogenic drivers, the pattern of tumor molecular heterogeneity, and the spread of the oncogenic events with metastatic PTC cells, all of the samples analyzed on each of the 41 PTC cases are shown. (**a**) Clinical-pathological features of the 41 patients including PTC subtype, histotype of each of the areas genotyped, sample type (pT, LNM, DM), age, sex, pT size, recurrence, timing of metastases (synchronous or metachronous), and survival status. Key colors are shown at the bottom of the figure. (**b**) Oncoprints of PTCs without DMs (left) and PTCs with DMs (right) including percentage of tumors altered on each driver. Key colors for genetic alterations found in the drivers investigated are shown boxed in the center of the figure. * *RAS* mutations are significantly correlated with PTCs with DMs. Fisher exact test two-tailed *P* = 0.003. # *TERT* amplification is significantly correlated with PTCs with DMs. Fisher exact test two-tailed *P* = 0.0448

The analysis of *TERT* gene copy number variations revealed the existence of *TERT* amplification (homozygous duplication/heterozygous triplication) in 7 PTCs (7/41 – 17%), mostly PTCs from the series of patients with DMs (30% PTCs with DMs vs. 5% PTCs without DMs) (Fig. 1).

*TPM* were detected in 18 PTCs (18/41 – 44%). More than half of the PTCs with DMs were mutated (55%), while among the PTCs without DMs, the prevalence was 33%. The mutants C228T and C250T were present in 78% and 22% of the mutated cases, respectively (Fig. 1).

Both events coexisted in 6 PTCs (6/41 – 15%), mostly PTCs from the series of patients with DMs (25% PTCs with DMs vs. 5% PTCs without DMs) (Fig. 1). *TERT* amplification was significantly more frequent in PTCs that harbored *TPM* (86%) than in wild type PTCs (14%) (*P* = 0.0313).

**Fig. 2:**
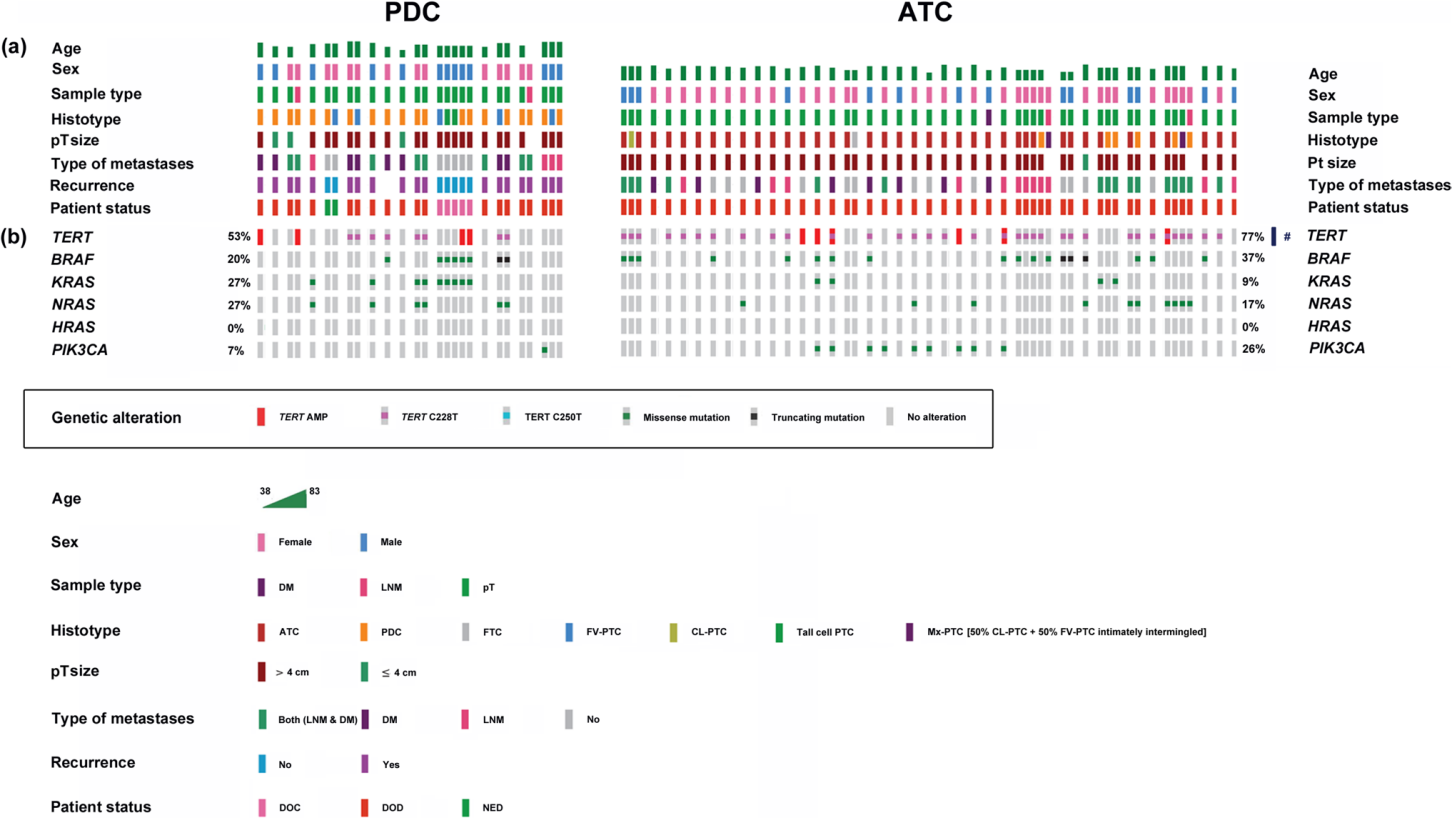
Pattern of oncogenic activation in 15 PDCs (left hand side of the figure) and 35 ATCs (right hand side of the figure). To facilitate the comprehension of the clonality or subclonality in the activation of the different oncogenic drivers, the pattern of tumor molecular heterogeneity, the segregation of the oncogenic events with tumor dedifferentiation within the pT, and the spread of the oncogenic events with metastatic cells, all of the samples analyzed on each of the 15 PDC and 35 ATC cases are shown. (**a**) Clinical-pathological features of the 50 patients including histotype of each of the areas genotyped, sample type (pT, LNM, DM), age, sex, pT size, type of metastatic spread (LNM, DM, or both), recurrence, and survival status. Key colors are shown at the bottom of the figure. (**b**) Oncoprints of PDCs (left) and ATCs (right) including percentage of tumors altered on each driver. Key colors for genetic alterations found in the drivers investigated are shown boxed in the center of the figure. # *TPM* are significantly correlated with ATCs. Fisher exact test two-tailed *P* = 0.0299

For comparison with PTC cases, the prevalence of *TERT* amplification and/or *TPM* was also evaluated in 50 aggressive thyroid cancers exhibiting a PDC (15 cases) or an ATC (35 cases) phenotype. A 20% of the PDCs and a 17% of the ATCs showed *TERT* amplification (Fig. 2). *TPM* were detected in 33% and 69% of the PDCs and ATCs, respectively. All the mutated PDCs and ATCs had the C228T mutation (Fig. 2). In contrast with PDCs, in which coexistence of *TPM* and *TERT* amplification was not seen, a 9% of the ATCs exhibited concurrence of both events (Fig. 2).

Figure 1 and Fig. 2 show the clinical-pathological features of the 41 PTCs, 15 PDCs, and 35 ATCs investigated as well as the distribution of *TERT* amplification and *TPM* as mechanisms of TERT re-expression and telomerase activation.

**Fig. 3:**
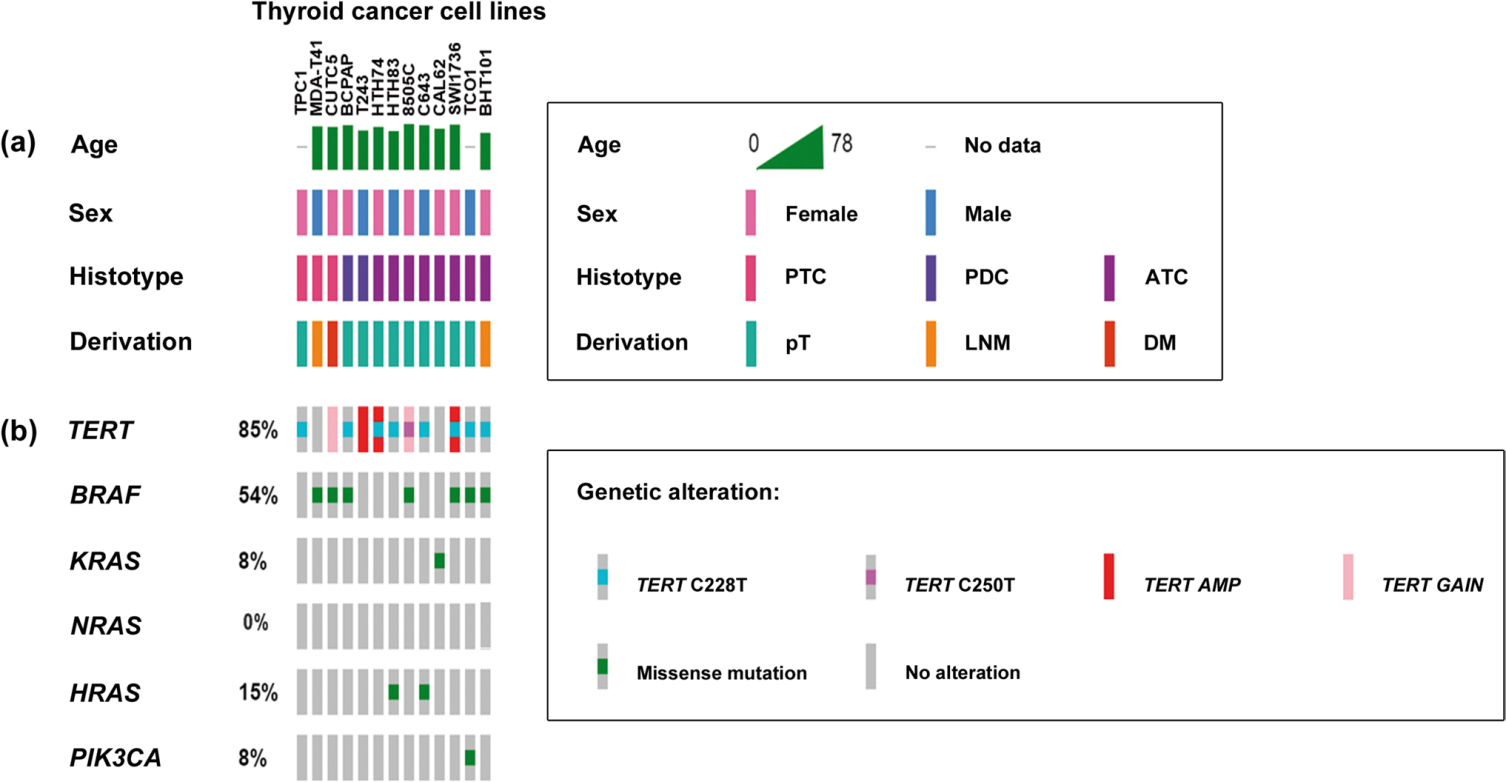
Pattern of oncogenic activation in 13 thyroid cancer cell lines derived from pTs, LNMs, or DMs showing tumors with different degrees of differentiation (PTC, PDC, or ATC). (**a**) Clinical-pathological features including age and sex of the patient bearing the tumor from which they were derived, histotype of the tumor from which they were generated (PTC, PDC, or ATC), and organ (pT, LNM, or DM) from which they were derived. Key colors are shown boxed in the upper right margin. (**b**) Oncoprint including percentage of cell lines altered on each driver. Key colors for genetic alterations found in the drivers investigated are shown boxed in the lower right margin

Likewise, *TERT* amplification was evaluated in 13 thyroid cancer cell lines derived from pTs, with different degrees of differentiation, or from DMs or LNMs of thyroid tumors. *TERT* amplification was found in the cell lines HTh74 and SW1736 derived from pTs of ATC phenotype and in the cell line T243 derived from a pT of PDC phenotype. A moderate increase in *TERT* copy number was observed in the cell line 8505C derived from a pT of ATC phenotype and in the cell line CUTC5 derived from a pleural metastasis of a PTC. See Fig. 3.

### Clonality of *TERT* activation events

#### *TERT* Promoter Mutations

More than one tumor area was characterized in 39 of the investigated PTCs (95%) (minimum 2 areas and maximum 10 areas).

If we take into account all the tumor samples analyzed in each PTC, regardless of the histotype observed in each area of the case under investigation and whether the sample corresponded to a pT, LNM, or DM, 50% of the cases with *TPM* were clonal, which means that the mutation was present in all the genotyped areas (Fig. 1).

When we evaluated the clonality or subclonality of *TPM* within the pT, the LNMs, or the DMs in PTC cases in which were analyzed more than one area of pT (41% PTCs), more than one LNM (36% PTCs), or more than one DM (15% PTCs), we found that clonality ranged between 63% in pTs and 75% in DMs. In 63% of the mutated PTCs with more than one area of pT analyzed, the mutation was clonal, mostly PTCs from the series of patients with DMs (4/5- 80%) (Fig. 1). In 50% of the mutated PTCs with more than one area of LNM analyzed, the mutation was clonal, was present in all the different LNMs screened (Fig. 1). In 75% of the mutated PTCs with more than one area of DM analyzed, the mutation was clonal, was present in all the different synchronous and/or metachronous DMs studied (Fig. 1).

In 50% of the PDCs with more than one genotyped pT area, the *TPM* analysis was positive and the mutation was clonal in all cases (100%) (Fig. 2). Likewise, in 71% of the ATCs with more than one area of pT genotyped, the *TPM* analysis was positive and the mutation was clonal in all cases (100%) (Fig. 2).

#### *TERT* Amplification

The analysis of clonality in PTCs with *TERT* amplification was somewhat more complex. If we take into account all the tumor samples analyzed in each of the 7 PTCs showing *TERT* amplification, regardless of the histotype observed in each area of the case under investigation and whether the sample corresponded to a pT, LNM, or DM, then 100% of the cases turned out to be subclonal. In none of the 7 PTCs was the amplification threshold reached in each and every one of the different areas that were genotyped in each case (Fig. 1). In 4 of the 7 cases (57%) showing *TERT* amplification, there was an increase in *TERT* gene copy number in all of the characterized areas, although in some areas, the increase in gene dosage was moderate or borderline. In the remaining 3 cases (43%), the increase in copy number was subclonal, as some of the genotyped areas were clearly diploid.

Different areas within the pT were analyzed in only 1 of the 3 PDCs bearing *TERT* amplification, and the amplification was circumscribed to the two phenotypically PDC areas, not being detected in areas of better differentiated phenotype (FV-PTC, Tall cell PTC) (Fig. 2). Similarly, in only 1 of the 6 ATCs bearing *TERT* amplification was analyzed more than 1 area of pT, and *TERT* amplification was restricted to the phenotypically ATC area (Fig. 2).

### Spread of *TERT* Activation Events with Metastatic PTC Cells and Tumor Dedifferentiation

The increase in *TERT* gene copy number spread in all 7 cases from pT to one or more of the analyzed metastatic niches. However, the increase in *TERT* gene dosage did not reach the amplification threshold in all the different tumor samples analyzed (pT, LNM, DM) in each of the cases investigated. A 67% of the PTCs showing *TERT* amplification at the pT revealed also *TERT* amplification in at least one of the metastatic niches genotyped (Fig. 1). In 2 cases (33%), the increase in *TERT* gene copy number found at the metastatic niche did not attain the threshold of amplification seen in the pT.

In 73% of the mutated PTCs with LNMs, the mutation spread from pT to LNMs. In 2 cases (18%), the *TPM* seemingly appeared de novo at the LNMs. In 1 case (9%), the *TPM* was limited to the pT. In all the mutated PTCs with more than one LNM genotyped, the *TPM* progressed to at least one of the LNMs (Fig. 1).

In 67% of the mutated PTCs with paired synchronous and/or metachronous DMs, the mutation spread from pT to DMs. In the remaining cases (33%), the mutation apparently emerged de novo at the DMs. In 75% of the mutated PTCs with more than one DM analyzed, the mutation spread from the pT to all the DMs. In one case, the mutation presumably originated de novo at the DMs (Fig. 1).

*TPM* were found in 46% of the PTCs in which LNMs and/or DMs were analyzed. The mutation spread from pTs to the metastatic niches in 67% of the mutated cases. In 5 cases (28%), the *TPM* seemingly appeared de novo at the LNMs and/or DMs, and in 1 case (6%), the *TPM* was limited to the pT.

If we take into account the type of *TPM* found, then the spread of the mutation with the metastatic cells was as follows. Half of the C250T mutants spread with metastatic PTC cells. The other half apparently originated de novo at the metastatic niche. The mutation C228T spread with metastatic PTC cells in 71% of the cases, was limited to the pT in 7% of the cases, and putatively originated de novo, at the metastatic niche, in 21% of the cases (Fig. 1).

A concurrent better differentiated area within the pT was investigated in 5 PDCs and 6 ATCs. *TERT* activation by *TPM* and/or *TERT* amplification was demonstrated in 60% of the PDCs and 67% of the ATCs in which a concurrent better differentiated area within the pT was genotyped. The mechanism of *TERT* activation evolved with tumor cell dedifferentiation in 67% of the PDCs and 100% of the ATCs (Fig. 2).

### Activation in PTCs of Other Oncogenic Drivers in Thyroid Carcinogenesis. Clonality and Spread with Metastatic PTC Cells

Figure 1 shows the clinical-pathological features of the 41 PTCs investigated as well as the distribution of *BRAF*, *RA*S (*H-*, *K-*, and *N-RAS*), and *PIK3CA* mutations.

See “online resource 2” for detailed information on the specific types of mutations found at *BRAF*, *RAS* (*H-*, *K-*, and *N-RAS*), and *PIK3CA*, as well as information regarding the clonal or subclonal nature of the mutations and their spread with metastatic PTC cells.

### Coexistence of *TERT* Activation by *TPM* and/or Amplification and Activation of Other Oncogenic Drivers. Tumor Molecular Heterogeneity in PTCs

Of the 30 PTCs with some genetic alteration, 18 (60%) demonstrated tumor molecular heterogeneity with several oncogenes concurrently activated (6 cases (33%) from the series of patients without DMs and 12 cases (67%) from the series of patients with DMs). See Fig. 1.

Two genes were concurrently activated in 12 of the 18 PTCs (67%) with molecular heterogeneity (5 of the 6 cases from the series without DMs (83%) and 7 of the 12 cases from the series with DMs (58%)) (Fig. 1). The most common association was activation of *TERT* by *TPM* and/or amplification and *BRAF* mutations, which was found in 8 PTCs (67%). Three genes were concurrently activated in 5 of the 18 PTCs (28%) with genetic heterogeneity (1 of the 6 cases from the series without DMs (17%) and 4 of the 12 cases from the series with DMs (33%)) (Fig. 1). Four genes concurrently activated were only seen in one case (6%) from the series of PTCs with DMs (Fig. 1).

Neither *BRAF*-like PTCs (18 cases) nor *RAS*-like PTCs (8 cases) mirrored the overall PTC cohort (41 cases) in terms of the significant association found between *TERT* amplification and *TPM*.

### Activation in PDCs and ATCs of Other Oncogenic Drivers in Thyroid Carcinogenesis. Tumor Molecular Heterogeneity

Figure 2 shows the clinical-pathological features of the 15 PDCs and 35 ATCs investigated as well as the distribution of *BRAF*, *RA*S (*H-*, *K-*, and *N-RAS*), and *PIK3CA* mutations.

See “online resource 2” for itemized information on the specific types of mutations found in both histotypes, the clonal or subclonal nature of the mutations, the transfer of mutations with tumor dedifferentiation within the pT, and tumor molecular heterogeneity.

### Relationship Between *TERT* Activation Patterns and Clinical-Pathological Parameters

To evaluate the clinical impact of the *TERT* activation patterns observed in the PTC group, *TERT* amplification and *TPM* showing separately or together, as well as in concurrence with other genetic events (*BRAF*, *RAS*, and *PIK3CA* mutations), were correlated with all of the prognostic clinical-pathological parameters summarized in Table 1. The results are shown in Table 2.

**Table 2:**
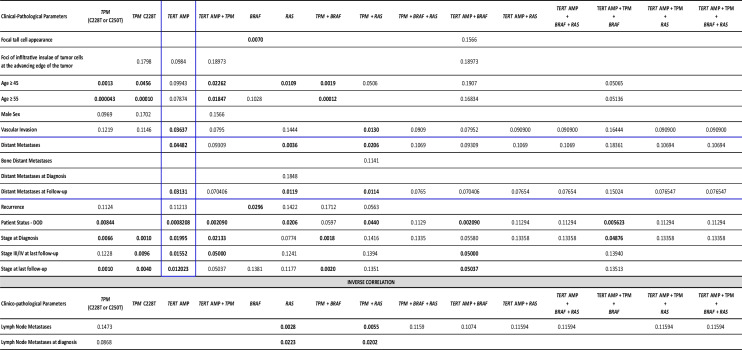
Relationship between *TERT* activation patterns and clinical-pathological parameters in papillary thyroid carcinomas

Only statistically significant correlations or trends for an association have been included in the table

Two tailed *P* values ≤ 0.05 (boldface) were considered statistically significant. *P* values between 0.05 and 0.18 were considered as a trend of correlation with the clinical-pathological trait

Abbreviations: *TPM* *TERT* promoter mutation, *TERT* *AMP* *TERT* amplification, *DOD *death of disease

Of note, *TERT* amplification appeared significantly correlated with vascular invasion (*P* = 0.0363), DMs at diagnosis and/or during the follow-up (*P* = 0.0448), DMs during the follow-up (*P* = 0.0313), tumor stage at diagnosis (*P* = 0.0199), tumor staging III/IV at last follow-up (*P* = 0.0155), and death of disease (DOD) status (*P* = 0.0008). The presence of foci of infiltrative insulae of tumor cells, surrounded by a prominent desmoplastic reaction at the advancing edge of the tumors, having an age at diagnosis ≥ of 45 or 55 years old, and tumor relapse revealed a meaningful trend for an association with *TERT* amplification (Table 2). The co-existence of *TERT* amplification with other investigated genetic events such as *TPM*, *BRAF* mutations, *RAS* mutations, or both *BRAF* + *RAS* mutations did not show any enhancing effect on the statistically significant associations already found between *TERT* amplification per se and different clinico-pathological parameters. Rather, the *P* value in most cases was no longer significant or worsened significantly. Only the tendency for a correlation with patient age ≥ 45 or ≥ 55 years old evolved to a significant linkage when *TERT* amplification coexisted with *TPM* (*P* = 0.0226 and *P* = 0.0184, respectively).

The occurrence of *TPM* only correlated significantly with older age at diagnosis (≥ 45 years [*P* = 0.0013] and ≥ 55 years [*P* = 0.00004]), tumor stage at diagnosis (*P* = 0.0066), tumor stage at last follow-up (*P* = 0.0010), and death of disease (DOD) status (*P* = 0.0084). Noteworthy, a significant association with vascular invasion (*P* = 0.0130), DMs at diagnosis and/or follow-up (*P* = 0.0206), and DMs at follow-up (*P* = 0.0114) showed up when *TPM* co-existed with *RAS* mutations. The co-occurrence of *TPM* with *BRAF* mutations or *BRAF* + *RAS* mutations did not show any improving effect on the statistically significant associations already found between *TPM* per se and different clinico-pathological features. Rather, the relationship with some features was no longer significant or worsened notably.

### Impact of *TERT* Activation Patterns on Clinical Course and Survival of PTC Patients

Follow-up information was available for all PTC patients analyzed in the study (Table 1). Tumor recurrence was observed in 23 PTC patients. PTC was the primary cause of death in 15 patients (DOD) (mean follow-up 137 months/11 years); 7 were AWD at the last follow-up (mean follow-up 174 months/14.5 years); and 18 were considered, after a life-long follow-up (mean follow-up 159 months/13 years), as having NED (Fig. 1). If we divide the casuistry investigated into cases without DMs (21 cases) and cases with DMs (20 cases), the mean follow-up in the first group was 141 months (12 years) and in the second group 159 months (13 years).

Seven (47%) of the patients who DOD exhibited *TERT* amplification, a genetic event that was not seen in any of the patients AWD or without evidence of disease. *TPM* were present in 11 (73%) of the patients DOD, 3 (43%) of the patients AWD, and 3 (17%) of the patients with NED. Both events (*TERT* amplification and *TPM*) coexisted in 40% of the patients who DOD and none of the patients that were AWD or were codified as NED (Fig. 1).

The probability of tumor recurrence was investigated in 34 patients (19 PTCs without DMs and 15 PTCs with DMs) with a follow-up period long enough to develop a true tumor recurrence. Of the 34 PTCs evaluated, 20 were positive (100% of cases with DMs (15 cases) and 26% of cases without DMs (5 cases)) and 14 were negative (74% of cases without DMs) (see Fig. 1). Of the 14 cases that did not show disease recurrence, none tested positive for *TERT* amplification or *TERT* amplification concurring with *TPM*, only two cases exhibited *TPM*. Of note, none of the latter 14 cases had signs of disease at the last follow-up (mean 145 months – 12 years). Among the 20 patients who developed recurrences in the follow-up, 11 finally died from the tumor, 5 were AWD, and 4 did not show any evidence of tumor after follow-up periods of 186 months (16 years), 138 months (12 years), and 206 months (17 years). While six of the 11 patients who developed recurrences and finally DOD demonstrated *TERT* amplification, none of the patients who developed tumor recurrences and were categorized as AWD or NED exhibited *TERT* amplification*.* Likewise, the coexistence of *TERT* amplification and *TPM* was seen in five of the 11 patients who developed recurrences and finally DOD, but in none of the patients with tumor recurrence who were AWD or showed no signs of tumor disease. *TPM* were found in 82% of the patients who recurred and ultimately died from the tumor, in 60% of the patients who recurred and were AWD at the last follow-up, and in 25% of the patients who recurred and had no evidence of disease at the last follow-up (Fig. 1).

**Fig. 4:**
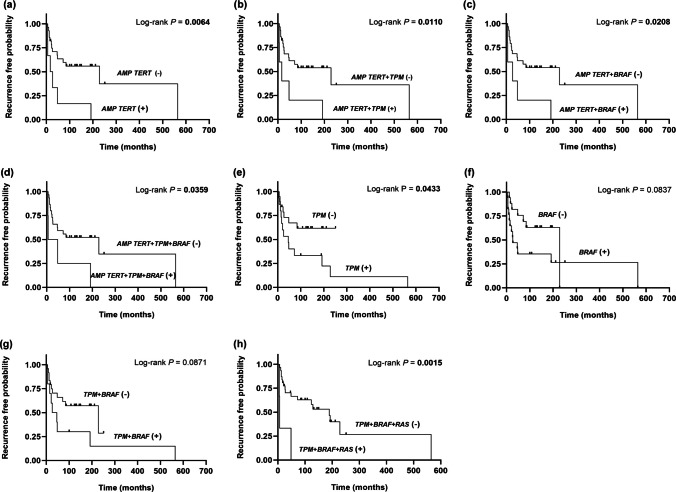
Impact of *TERT* activation patterns on disease-related recurrence. Kaplan–Meier estimate of recurrence-free probability in PTC patients with information available regarding the exact moment of tumor relapse. Patients were dichotomized according to (**a**) the presence of *TERT* amplification; (**b**) the concurrence of *TERT* amplification and *TPM*; (**c**) the concurrence of *TERT* amplification and *BRAF* mutations; (**d**) the concurrence of *TERT* amplification and *TPM* and *BRAF* mutations; (**e**) the existence of *TPM*; (**f**) the presence of *BRAF* mutations; (**g**) the coexistence of *TPM* and *BRAF* mutations; and (**h**) the coexistence of *TPM* and *BRAF* mutations and *RAS* mutations

Figure 4 and Table 3 show the most relevant results of the Kaplan–Meier analysis in the 34 PTC patients in whom the risk of tumor recurrence associated with the presence of different genetic events studied in the tumors was evaluated. When the impact on tumor relapse was evaluated in the 19 PTC patients without DMs, the probability of tumor recurrence appeared significantly associated with the presence of *BRAF* mutations and the coexistence of *BRAF* mutations and *TPM* (Log-rank *P* = 0.0087 and Log-rank *P* = 0.0282, respectively) (“Online resource – Figure-1”). None of the 19 cases without DMs that recurred exhibited *TERT* amplification and the existence of *TPM* revealed only a trend of association with tumor relapse (Log-rank *P* = 0.0914) (“Online resource – Figure-1”).

**Table 3:** Impact of *TERT* activation patterns on disease-related recurrence and survival in papillary thyroid carcinomas

Kaplan - Meier estimates analysis - *P* values			
Mechanism inmortalization	Genetic event	Recurrence	Survival
*TERT* *AMP*	*** TERT AMP***	**0.0064**	** < 0.0001**
	*** TERT AMP * + *TPM***	**0.0110**	** < 0.0001**
	*** TERT AMP * + *BRAF***	**0.0208**	**0.0003**
	*** TERT AMP * + *RAS***	0.1739	0.0668
	*** TERT AMP * + *PIK3CA***	NA	**0.0004**
*TPM*	***TPM***	**0.0433**	**0.0410**
	***TPM* + *BRAF***	0.0871	0.0752
	***TPM* + *RAS***	NS	NS
	***TPM* + *PIK3CA***	0.0932	0.1844
*TERT* *AMP* + *TPM*	*** TERT AMP * + *TPM***	**0.0110**	** < 0.0001**
	*** TERT AMP * + *TPM* + *BRAF***	**0.0359**	**0.0010**
	*** TERT AMP * + *TPM* + *RAS***^**a**^	0.1739	0.0668
	*** TERT AMP * + *TPM* + *PIK3CA***	NA	**0.0004**
*TPM* + other (*BRAF* or *RAS* or *PIK3CA*)	***TPM* + *BRAF* + *RAS***	**0.0015**	0.0668
	***TPM* + *BRAF* + *PIK3CA***^**b**^	0.0932	0.1844
	***TPM* + *RAS* + *PIK3CA***	NA	NA

Only statistically significant correlations or trends for an association have been included in the table. *P* values ≤ 0.05 (boldface) were considered statistically significant. *P* values between 0.05 and 0.18 were considered as a trend of correlation

Abbreviations: *TPM* *TERT* promoter mutation, *TERT AMP* *TERT* amplification, *NS* Log-rank analysis not significative, *NA* not applicable because no single case met the genetic criteria

^a^Same *P* values as *TERT* *AMP* + *RAS*

^b^Same *P* values as *TPM* + *PIK3CA*

**Fig. 5:**
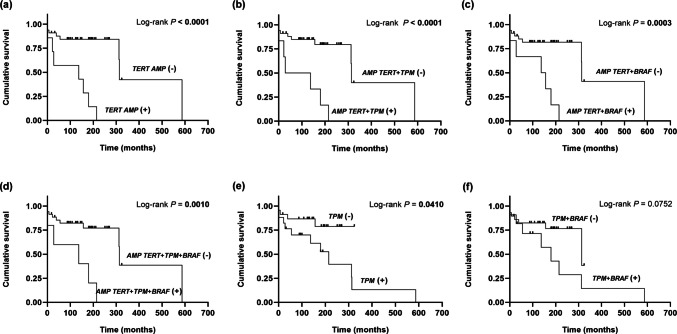
Impact of *TERT* activation patterns on disease-related survival. Kaplan–Meier estimate of likelihood of disease-related death in PTC patients with information available regarding the exact moment of DOD. Patients were dichotomized according to (**a**) the presence of *TERT* amplification; (**b**) the concurrence of *TERT* amplification and *TPM*; (**c**) the concurrence of *TERT* amplification and *BRAF* mutations; (**d**) the concurrence of *TERT* amplification and *TPM* and *BRAF* mutations; (**e**) the existence of *TPM*; and (**f**) the coexistence of *TPM* and *BRAF* mutations

Disease-specific survival was evaluated in 40 cases (20 patients without DMs and 20 patients with DMs). Figure 5 and Table 3 illustrate the most relevant genetic events studied in the 40 tumors, which were found to correlate with poor survival. No significant correlation with disease specific survival was found with the presence of *BRAF* mutations or *RAS* mutations alone or in coexistence with *TPM*. When the analysis of tumor-related death was approached in the 20 cases without DMs, *TERT* amplification also emerged as a better predictor of reduced survival than *TPM* (Log-rank *P* < 0.0001 and Log-rank *P* = 0.0140, respectively) (“Online resource – Figure-2”). *BRAF* mutations per se only exhibited a trend of correlation with poor survival (Log-rank *P* = 0.1451), but when concurring with *TPM* the likelihood of tumor-related death increased significantly (Log-rank *P* = 0.0066) (“Online resource – Figure-2”).

Table 4 summarizes those clinical-pathological and genetic variables that in the univariate analysis of the PTC patients investigated in this study showed a strong tendency towards an association with tumor recurrence or death or were significantly correlated with tumor-related recurrence or death. Clinical-pathological parameters that have been previously shown to be associated with a worse prognosis were corroborated as statistically significant poor prognostic indicators in our series. The presence of foci of infiltrative insulae of tumor cells, surrounded by a desmoplastic reaction at the advancing edge of the tumors, and the presence of focal tall cell appearance were also significant predictors of tumor relapse (RR = 3.442, *P* = 0.019 and RR = 5.853, *P* = 0.0005, respectively). Of note, *TERT* amplification increased the likelihood of tumor recurrence (RR = 3.656, *P* = 0.010) and the risk of death of disease (RR = 7.930, *P* = 0.0004). Univariate analyses also revealed a significantly higher likelihood of tumor relapse or tumor-related death among patients with PTCs whose cells showed *TERT* amplification concurring with other genetic alterations such as *TPM*, *BRAF*, or *TPM* + *BRAF* mutations (see Table 4).

**Table 4:** Univariate analysis - relative risk and likelihood ratio of disease-related recurrence or death associated with clinical-pathological parameters or altered genotype

Cox proportional hazards model and Likelihood ratio test					
	RR/HR	95% CI	*P* value	Likelihood ratio	*P* value
**TUMOR RECURRENCE**					
**Clinical-pathological variables**					
Foci of infiltrative insulae of tumor cells at the advancing edge of the tumor	**3.442**	**1.215–9.754**	**0.0199**	**6.138**	**0.0132**
Focal tall cell appearance	**5.853**	**2.163–15.841**	**0.0005**	**11.409 **	**0.0007**
Age ≥ 55	**0.197**	**0.072–0.538**	**0.0015**	**10.115 **	**0.0014**
Vascular invasion	1.943	0.760–4.968	0.1653	2.002	0.1570
Distant metastases at diagnosis	**3.786**	**1.357–10.558**	**0.0109**	**5.548**	**0.0184**
Stage at diagnosis I–II vs. III–IV	5.744	0.475–69.340	0.169	**11.829 **	**0.0079**
**Genotype variables**					
*TERT AMP*	**3.656**	**1.357–9.850**	**0.0103**	**5.498**	**0.0190**
*TERT AMP* + *TPM*	**3.574**	**1.264–10.098**	**0.0162**	**4.660**	**0.0308**
*TERT AMP* + *BRAF*	**3.223**	**1.140–9.109**	**0.0272**	**4.009**	**0.0452**
*TPM*	2.544	0.993–6.515	0.0515	**3.965**	**0.0464**
*TPM* + *BRAF*	2.182	0.872–5.458	0.0951	2.618	0.1056
*TERT AMP* + *TPM* + *BRAF*	**3.162**	**1.034–9.672**	**0.0434**	**3.253**	**0.0712**
*BRAF*	2.250	0.877–5.774	0.0914	2.973	0.0846
*BRAF* + OTHER* (TPM * and/or *RAS* and/or* PI3K)*	2.182	0.872–5.458	0.0951	2.618	0.1056
**SURVIVAL**					
**Clinical-pathological variables**					
Vascular invasion	**4.814**	**1.330**–**17.420**	**0.0165**	**7.140**	**0.0075**
Extrathyroidal extension	**4.146**	**1.044**–**16.457**	**0.0431**	**4.568**	**0.0325**
Distant metastases at diagnosis	**5.673**	**1.719**–**18.722**	**0.0043**	**7.635**	**0.0057**
Stage at diagnosis I–II vs. III–IV	**9.377**	**2.671**–**32.913**	**0.0004**	**12.451 **	**0.00041**
**Genotype variables**					
*TERT AMP*	**7.930**	**2.503**–**25.120**	**0.0004**	**11.798 **	**0.0005**
*TERT AMP* + *TPM*	**7.088**	**2.276**–**22.072**	**0.0007**	**9.982**	**0.0015**
*TERT AMP* + *BRAF*	**6.145**	**1.975**–**19.119**	**0.0017**	**8.734**	**0.0031**
*TERT AMP* + *RAS*	3.158	0.850–11.728	0.0857	2.391	0.1220
*TERT AMP* + *BRAF* + *RAS*	3.158	0.850–11.728	0.0857	2.391	0.1220
*TPM*	3.200	0.988–10.361	0.0523	**4.196**	**0.0405**
*TPM* + *BRAF*	2.525	0.875–7.279	0.0863	2.841	0.0918
*TERT AMP* + *TPM* + *BRAF*	**5.582**	**1.768**–**17.619**	**0.0033**	**7.137**	**0.0075**
*TERT AMP* + *TPM* + *RAS*	3.158	0.850–11.728	0.0857	2.391	0.1220
*TERT AMP* + *TPM* + *BRAF* + *RAS*	3.158	0.850–11.728	0.0857	2.391	0.1220
*TPM* + *BRAF* + *RAS*	3.158	0.850–11.728	0.0857	2.391	0.1220
*PIK3CA*	**6.902**	**1.359**–**35.0544**	**0.0197**	**3.825**	**0.0504**
*BRAF* + OTHER* (TPM * and/or *RAS* and/or *PI3K*)	2.345	0.806–6.820	0.1176	2.471	0.1158

Only those variables found significantly associated or exhibiting a trend of correlation with disease-related recurrence or death are shown

*P* values ≤ 0.05 (boldface) were considered statistically significant. *P* values between 0.05 and 0.12 were considered as a trend of correlation

Abbreviations: *TPM* *TERT* promoter mutation, *TERT AMP* *TERT* amplification, *RR/HR* relative risk/hazard ratio, *95% CI* 95% confidence interval

The Akaike Information Criterion (**AIC**) was used in Cox proportional hazards regression modeling to find the simplest and most parsimonious multivariate model, which best explains the dependent variable time elapsed until recurrence or survival (see Table 5). The final, unique, selected multivariate model for recurrence and survival includes all those variables, among all possible explanatory variables initially considered, that contribute the most to explain time to recurrence or death, which implies, independently of the *P* value, their relevance to survival or event risk. The analysis of tumor recurrence revealed that *TERT* amplification was a better predictor of tumor relapse than *TPM*, extrathyroidal extension, tumor multifocality or tumor size ≥ 5 cm, DMs at diagnosis, and stage at diagnosis I–II vs. III–IV. Furthermore, *TERT* amplification predicted tumor relapse independently of other variables that also exhibited predictive value such as the presence of foci of infiltrative insulae of tumor cells, surrounded by a desmoplastic reaction at the advancing edge of the tumor, areas of focal tall cell appearance, age ≥ 55 years old, male sex, vascular invasion, LNMs at diagnosis, and *BRAF* mutations. In contrast to *TERT* amplification, the presence of *BRAF* mutations had a protective effect on tumor recurrence (RR/HR 0.154). PTCs whose cells had *TERT* amplification would have a relative risk of developing a recurrence 5.405 times higher than PTCs whose cells did not have *TERT* amplification. Likewise, the multivariate analysis of tumor-related death showed that *TERT* amplification was a better predictor of survival than *TPM*, male sex, tumor multifocality or tumor size ≥ 5 cm, vascular invasion, DMs at diagnosis and/or follow-up, and stage at diagnosis I–II vs. III–IV. Even more, *TERT* amplification was able to prognosticate a poor survival independently of other variables that also exhibited predictive value such as age ≥ 55 years old, extrathyroidal extension, and *BRAF* mutations. Similarly to what have seen in tumor recurrence, the presence of *BRAF* mutations on PTC cells decreased de risk of dying due to the tumor (RR/HR 0.333). It had an opposite effect to *TERT* amplification. In PTCs with *TERT* amplification, the likelihood of poor survival was 7.389 greater than in PTCs without *TERT* amplification.

**Table 5:** Multivariate analysis - relative risk of disease-related recurrence and death according to clinical-pathological and genotype indicators. Cox proportional hazards regression modeling using the Akaike Information Criterion (AIC) - Wald test - Likelihood ratio test

Variables	RR/HR	95% CI	*P* value	Wald test	*P* value	Likelihood ratio	*P* value
**RECURRENCE**^d,#^							
*TERT AMP*	5.405	1.132–25.792	0.0343	18.970	0.01504	37.072	0.00001
*BRAF*	0.154	0.026–0.915	0.0397				
Age ≥ 55	8.514	2.040–35.531	0.0033				
Male sex	5.390	1.126–25.786	0.0349				
Vascular invasion	0.095	0.016–0.562	0.0094				
Lymph node metastases at diagnosis	0.090	0.014–0.576	0.0110				
Foci infiltrative ITC at advancing edge of tumor	11.356	1.483–86.947	0.0193				
Focal tall cell appearance	24.972	3.249–191.926	0.0019				
**SURVIVAL**^d,#^							
*TERT AMP*	7.389	1.363–40.041	0.0203	19.180	0.00072	26.827	0.00002
*BRAF*	0.333	0.077–1.433	0.1399*				
Age ≥ 55	9.615	1.655–55.862	0.0117				
Extrathyroidal extension	4.448	0.796–24.842	0.0889*				

Abbreviations:* TERT AMP* *TERT * amplification, *ITC* insulae of tumor cells, *RR/HR* relative risk/hazard ratio, *95% CI* 95% confidence interval

^d^First, a complete model is built, which contains all the possible predictor/explanatory/independent variables that, both in the series under study and in the published literature, have been shown that may significantly impact on PTC relapse or death. In the case of tumor recurrence, the initial model included the following variables: *TERT AMP*, *TPM*, *BRAF* mutations, age ≥ 55 years old, male sex, multifocality or tumor size ≥ 5 cm, extrathyroidal extension, vascular invasion, focal tall cell appearance, foci of infiltrative insulae of tumor cells, surrounded by a desmoplastic reaction at the advancing edge of the tumor, lymph node metastases at diagnosis, distant metastases at diagnosis, and stage at diagnosis I–II vs. III–IV. In the case of survival, the initial model included the following variables: *TERT AMP*, *TPM*, *BRAF* mutations, age ≥ 55 years old, male sex, multifocality or tumor size ≥ 5 cm, extrathyroidal extension, vascular invasion, distant metastases at diagnosis and/or follow-up, and stage at diagnosis I–II vs. III–IV. Next, from this complete models, a narrow down of variables is performed, eliminating those that do not have predictive capacity. This filtering is carried out both in forward and backward directions following the Akaike Information Criterion (AIC)

^#^The Akaike Information Criterion (AIC) selected, for both recurrence and survival, the best, simplest, and most parsimonious multivariate model, balancing model complexity and goodness of fit. The final selected multivariate model includes all those variables, among all possible explanatory variables initially considered, that contribute the most to explain “time to an event” (recurrence or survival), which implies, independently of the *P* value, their relevance to survival or event risk

^*^Variables that independently of the *P* values shown in the table are considered following the AIC as predictors of survival

## Discussion

Studies focused on identifying and setting out all the different molecular events involved in the early stages of the immortalization process that refine the prognostic value of known markers are needed. The *TERT* locus is a critical vulnerability site for tumor progression, dedifferentiation, and aggressiveness in thyroid carcinogenesis. In addition to *TPM*, the alterations that may result in TERT re-expression and telomerase activation in thyroid cancer cells include genomic amplification as well as structural alterations at the *TERT* locus, chromatin remodeling, or hypermethylation events upstream of the *TERT* transcriptional start site [15]. Most of those mechanisms, which might significantly impact on telomerase function in thyroid cancer cells, are not fully understood because most of the research efforts so far have concentrated on the analysis of *TPM* [16–20]. Since 2014, when the TCGA research network approached the genomic characterization of PTCs [5], very few original research studies have focused on investigating the prevalence of somatic *TERT* CNV (copy number gains or amplification) as a mechanism of telomerase reactivation in PTCs [7–9].

In this study, we focused on *TERT* amplification as an alternative mechanism to *TPM* for TERT re-expression and telomerase activation. The overall prevalence of *TERT* amplification found among PTCs, PDCs, and ATCs was 17%, 20%, and 17%, respectively. In agreement with the theory that considers the molecular alterations involved in TERT re-expression and telomerase activation as events associated with advanced stage, clinically aggressive carcinomas, in our study, *TERT* amplification was much more frequent in PTCs with DMs. An 86% of the PTCs bearing *TERT* amplification were from the subset of patients with DMs. The prevalence among PTCs with paired LNMs that did not develop DMs at diagnosis and/or during the follow-up was similar to that seen in the conventional, low/intermediate risk PTCs without DMs genotyped by the TCGA research network (4.7% this study vs. 4.4% TCGA study) [5].

The study of 13 thyroid cancer cell lines revealed, for the first time, in vitro study models that reproduced the results found in tumor samples.

In the TCGA study, arm level alterations occurred more frequently in FV-PTCs than in CL-PTCs (*P* < 0.008). Unsupervised clustering of chromosomal arm-level alterations defined 4 distinct groups of PTCs, one of which was characterized by a higher frequency of focal somatic CNV (gains and losses) and was enriched for FV-PTCs. Copy gains/amplifications at 5p (*TERT*) were found in 3.4% of the CL-PTCs, 9.5% of the FV-PTCs, and 2.85% of the tall cell PTCs analyzed by the TCGA [5]. Likewise, we have also found that *TERT* amplification was more frequent among the FV-PTCs (21.05% of the total cases; 57.14% of the cases with amplification. All 4 cases were infiltrative FV-PTCs) than among CL-PTCs (13.63% of the total cases; 43% of the cases with amplification). In our series, however, we did not see a significant correlation with the follicular variant tumor subtype. In 2016, Yoo SK and colleagues also found a higher percentage of somatic arm-level CNV in FV-PTCs than in CL-PTCs [21]. In the past, it has been hypothesized that CNV and chromosomal instability may drive FV-PTCs and pathogenesis, with CNV leading to a different tumor histology [22].

To verify the concurrent or mutually exclusive nature of *TERT* amplification and *TPM* and the alleged association of *TERT* amplification with other oncogenic drivers in thyroid carcinogenesis, we investigated in the same cohort of tumors the presence of *TPM*, *BRAF*, *H-RAS*, *K-RAS*, *N-RAS*, and *PIK3CA* mutations. In contrast with Gupta’s findings [8] and similarly to results of other authors [6, 7, 9, 10], in our study, *TERT* amplification and *TPM* co-occurred in 15%, 0%, and 9% of the PTCs, PDCs, and ATCs, respectively. An 83% of the PTCs bearing both events were from the subset of patients with DMs. In PTCs, but not in PDCs and ATCs, *TERT* amplification and *TPM* were significantly correlated (*P* = 0.0313). It has been shown that TERT expression is significantly higher in tumors with *TERT* amplification than in tumors without *TERT* amplification (*P* = 0.04), as well as that the combination of *TERT* amplification and *TPM* further increases the expression [5]. It appears that the two events might cooperate in TERT reactivation and thyroid cancer progression. Barthel et al. showed that the highest telomerase activity is found in tumors with *TERT* amplification [2]. When genome data sets of different types of cancers (lung squamous cell carcinoma, bladder urothelial carcinoma, metastatic melanoma, and hepatocellular carcinoma), with information on TERT mRNA expression and copy number alterations, are uploaded to cBioPortal (http://cbioportal.org), we see that *TERT* amplification is associated with much higher expression of TERT than *TERT* copy number gains (see “online resource 3” for detailed information on TERT mRNA expression analyses in different tumors using the web resource cBioPortal for exploring, visualizing, and analyzing multidimensional cancer genomics data sets). In 2021, Gupta S et al. also corroborated that those thyroid tumors with genomic *TERT* amplification and rearrangements exhibited statistically significant higher increases in *TERT* expression than tumors with *TPM* [8]. In our series, the lack of frozen tissue from any of the samples analyzed hampered the correlation between *TERT* amplification and TERT mRNA expression levels. Considering the different analyses we carried out in cBioPortal and the rest of the research studies mentioned, one would expect that the cases with *TERT* amplification in our case series would behave with respect to TERT expression in a similar way to those cases reported in the literature that exhibited only *TERT* amplification or *TERT* amplification plus *TPM*. On the other hand, TERT immunohistochemistry (IHQ) or measurement of protein expression did not work properly (data not shown). Curiously, in a study by Paulson et al. in thyroid cancer, no correlation between TERT mRNA expression and TERT IHQ could be demonstrated [23]. Although several studies have attempted to evaluate TERT protein expression using IHQ, this has been controversial. The efforts have been hindered by poor reproducibility, unexpected patterns of subcellular localization, and documented cross-reactivity with other proteins [8, 24, 25]. Thus far, TERT IHQ does not seem to be a useful clinical tool for prognostication [23, 26]. In PTCs, no correlation has been reported between TERT IHQ and clinical-pathological traits [26].

Coexistence of *TERT* amplification and *BRAF* mutations was seen in 15%, 7%, and 9% of the PTCs, PDCs, and ATCs, respectively. Only in PTCs was found a trend of correlation between *TERT* amplification and *BRAF* mutations (*P* = 0.0994). Coexistence of *TERT* amplification and *RAS* mutations was seen in 7%, 7%, and 9% of the PTCs, PDCs, and ATCs, respectively. When the three histotypes investigated (PTC, PDC, and ATC) were analyzed together, a trend for an association between *TERT* amplification and *RAS* mutations appeared (*P* = 0.1166). Coexistence of *TERT* amplification and *PIK3CA* mutations was seen in 2%, 0%, and 11% of the PTCs, PDCs, and ATCs, respectively. *TERT* amplification and *PIK3CA* mutations were significantly correlated (*P* = 0.0272) in ATC.

While in the TCGA study somatic CNV were significantly enriched in cases with no driver mutation, suggesting that somatic CNV may also drive PTC, in our casuistry only three cases (2 PDCs and 1 ATC) did not exhibit any of the other genetic drivers screened. Most of the cases had 2 or 3 associated mutational events. A putative explanation for this difference is that while in the TCGA study were mainly characterized conventional, low/intermediate-risk PTCs without DMs (98,4%), in our casuistry, have been mainly characterized advanced stage, clinically aggressive cancers (77%). It is known that advanced stage, clinically aggressive tumors are prone to genomic instability and accumulation of different genetic events. Nonetheless, the 5 PTCs with aggressive histology genotyped in the TCGA study revealed, as did the PTCs with DMs analyzed in this study, a much higher mutational burden than conventional low/intermediate-risk PTCs [5]. When the pTs grow slowly, the growth advantage of additional driver mutations is larger, subclones greatly expand, and the rate of mutations in driver genes within the pTs before their clinical detection increases.

Molecular heterogeneity was found in 60% of the PTCs, 60% of the PDCs, and 66% of the ATCs genotyped (see Fig. 1 and Fig. 2). More than half of the PTCs (67%) exhibiting molecular heterogeneity were PTCs with DMs. Two oncogenes were concurrently activated in 67%, 33%, and 58% of the PTCs, PDCs, and ATCs, respectively. Three oncogenes coexisted in 28%, 67%, and 32% of the PTCs, PDCs, and ATCs, respectively. Four oncogenes activated were seen in 6%, 0%, and 11% of the PTCs, PDCs, and ATCs, respectively*. TERT* activation may predispose, through the induction of genomic and chromosomal instability, to the acquisition of secondary genetic events. Additional molecular alterations, which, in turn, may activate signaling pathways that account for the aggressiveness of some PTCs.

Driver gene mutation heterogeneity within primary PTCs and matched DMs is commonly ignored in the clinical setting leading to an inadequate risk-based stratification of patients, improper clinical surveillance, and erroneous therapeutical planning.

To date, none of the few published studies on *TERT* amplification in PTCs has addressed the clonal or non-clonal nature of *TERT* activation by *TERT* amplification or by *TPM*, in the case of having analyzed both events.

In our study, we observed that in PTCs *TERT* amplification was a subclonal event. Although in more than half (57%) of the PTCs showing variations in *TERT* gene dosage was found an increase in *TERT* copy number in all the tumor samples analyzed in each case, not in all of the areas genotyped in those cases the increase reached the established amplification threshold. Watkins TB et al. have demonstrated that continuous chromosomal instability results in pervasive somatic CNV heterogeneity. Using a multi-sample phasing in the analysis of somatic CNV across 22 tumor types showed that focal *TERT* amplifications were frequently subclonal [27]. In PDCs and ATCs, when concurrent better differentiated areas within the pT were genotyped, we saw that *TERT* amplification appeared to show a preference for the phenotypically less differentiated areas.

In our study, *TPM* were found to be clonal in half of the mutated PTCs. When clonality was assessed within the pTs of the mutated PTCs, *TPM* were clonal in more than half of the cases (63%), mostly advanced PTCs with DMs. Within the LNMs and DMs, clonality reached levels of 50% and 75%, respectively. Regardless of whether the DMs were synchronous or metachronous, *TPM* tended to be present in all of the different DMs screened. The increase in the rate of *TPM* clonality at DMs appears to indicate that the *TERT*-mutated bearing cell clusters that extravasate and reach the metastatic sites do not experience serious constraints for survival and homing. It might occur a clonal overgrowth of cells with *TPM* at DMs, meaning that cells with *TPM*, which are known to have a growth advantage, overgrow all other cells. Among PDCs and ATCs, clonality was only investigated within pTs, observing in both histotypes that *TPM* were clonal. Our data suggest that there is a trend towards clonality of *TPM* with tumor progression, dedifferentiation, and clinical aggressiveness (DMs), which is consistent with previous findings reported by Landa et al. on *TPM* prevalence and clonality in thyroid cancer [28]. Landa et al. found that *TPM* were scarce and subclonal in conventional PTCs and highly prevalent and clonal in the more aggressive types of thyroid cancer (PDC and ATC). Similarly, the genotyping of 355 PTCs by Liang et al. revealed that *TPM* occurred in a subclonal manner [29].

Our results regarding the clonal nature of *TERT* activation events clearly illustrate that when genotyping is restricted to only one area, primarily of pT or LNM and less crucially of DM, it can occur that subclonal *TERT* amplification or *TPM* may evade detection. It has been shown, in different tumor types, that genetic analyses from single biopsies may lead to an underestimation of the complex mutational portraits that characterize different advance stage tumors [30–33]. Another detail to have in consideration when analyzing the putative subclonality of a mutational event is the sensitivity of the methodology applied to detect mutant alleles. It has been demonstrated, in PTCs and follicular thyroid tumors of uncertain malignant potential, that digital droplet PCR (ddPCR) has a higher sensitivity for detecting *TPM* in samples that have very low mutant allele frequencies [34, 35]. The latter means that we might have missed some *TPM* and, thus, the clonality observed in the whole series of PTC could be higher than the reported of 50%.

To know the clonal status of different genetic events within a particular tumor it is crucial for the success of personalized medicine. Targeting of subclonal events will be certainly insufficient to prevent tumor progression. Indeed, inhibition of subclonal alterations will most probably only cause the outgrowth of other untargeted mutated cancer cells that drive tumor progression, as well as the failure of targeted therapies and a fatal outcome [36]. The commonest is that clinicians make a decision in favor a particular targeted therapy on the basis of a single biopsy of the pT, which may not reflect the clonal status of the targeted gene, a mistaken strategy that facilitates the appearance of therapy resistance.

Heretofore, our study has screened the largest series of primary PTCs with paired LNMs (30 cases) and primary PTCs with matching synchronous and/or metachronous DMs (20 cases). Near 70% of the PTCs showing *TERT* amplification at the pT revealed also *TERT* amplification in at least one of the metastatic niches genotyped. *TPM* were found to spread with metastatic PTC cells to LNMs or DMs in 73% and 67% of the cases, respectively. Apparently de novo mutations at LNMs or DMs were seen in 18% and 33% of the PTCs, respectively. In most of the mutated cases, the metastatic PTC cells maintained their primary mutational traits after metastases development, with limited influence of the metastatic niche. In some of the mutated cases, however, regional pressures, presumably exerted by the homing microenvironment at metastases, could have led to the appearance of metastatic tumor cells bearing advantageous de novo/private mutations. The more common C228T *TPM* exhibited a greater propensity to spread from the pT to metastases, while the less common C250T *TPM* seemingly originated more frequently de novo, at the metastases. It is tempting to speculate that the C250T *TPM* is prone to emerge in metastatic milieus, contributing not only to shape the metastases but also the appearance of resistance to targeted therapies. It could also be that the mutations considered as de novo mutations in LNMs and DMs are not such. We cannot rule out the possibility of having lost subclonal *TPM* in pTs. Mutations present in a small number of pT cells may evade detection. It has been shown in PTCs that *TPM* in samples that harbor very low mutant allele frequencies can be missed by sanger sequencing [35].

*TERT* activation by *TERT* amplification and/or *TPM* spread from the better differentiated area to the less differentiated area within the pT in two-thirds of the PDCs and 100% of the ATCs. Despite the limited number of PDCs and ATCs in which it was possible to investigate the transfer of the *TERT *activation mechanism from a better differentiated area to a less differentiated area within the pT, it is tempting to hypothesize that once *TERT* is activated it is prone to evolve with tumor cell dedifferentiation. The dissemination of *TERT* amplification with cellular dedifferentiation represents additional molecular evidence for a stepwise progression from PTC to PDC and ATC within a multistage genetic model of thyroid follicular cell tumorigenesis, but does not necessarily mean that it is the driving force that underlies histological dedifferentiation. The overall prevalence of *TERT* amplification in PDCs and ATCs, seen in this study and previous studies, is lower or similar to that demonstrated for other oncogenes, which implies that it is not a dominant event in the pathogenesis of PDCs and ATCs.

Up till now, none of the few studies performed on *TERT* amplification as a mechanism of TERT re-expression and telomerase activation in thyroid cancer [5–9] has appraised the impact of *TERT* amplification on the clinical course (recurrence and survival) of PTC patients. Only Paulsson et al. have assessed the impact of *TERT* aberrancies, including *TERT* copy number gains, in tumor-related relapse, but the tumors investigated were FTCs not PTCs [6]. To the best of our knowledge, this is the first study that evaluates the relationship between *TERT* amplification in PTCs and clinical-pathological parameters of poor prognosis, recurrence, and survival. Some of the previous studies concur in considering *TERT* amplification, like *TPM*, as a late event in thyroid carcinogenesis, more common in advanced tumor stages. None of those studies has proved, however, a statistically significant correlation between *TERT* amplification and the patient’s tumor stage. Importantly, some of these studies also agree on the need to analyze larger series of carcinomas in advanced stages, aimed at defining the impact that pathogenic alterations of *TERT*, different from *TPM*, have on the prognosis of patients [7–9].

Our analysis of PTC patients reveals that *TERT* amplification, as shown in Table 2, has an enormous impact on the clinical course of PTC patients. The statistically significant associations that we found with vascular invasion, DMs at diagnosis and/or during follow-up, and metachronous DMs, which develop during the patient’s follow-up, are those that can have a greatest impact on patient’s prognosis. Based on our results, *TERT* amplification is a major determinant of the metastatic capability of PTCs, increasing the risk of DMs at diagnosis or during the follow-up. De facto, when *TERT* amplification was present in PTC cells, a significant relationship was observed with tumor stage at diagnosis, stage III/IV at last follow-up, and a DOD patient status. Our findings are consistent with reported data showing that *TERT*, independently of its function in maintaining telomere length, participates in the activation of the epithelial-mesenchymal transition (EMT), which implies the induction in cells of migratory and invasive capacities. TERT interacts with β-catenin (one of the EMT-associated transcription factors) and together associate with mesenchymal marker promoters to drive their expression [37]. Moreover, it has been demonstrated in highly metastatic PTCs that CDH6 expression, a class II cadherin aberrantly reactivated in cancer, is restricted to EMT cells that also exhibit a higher incidence of *TERT* amplification, a finding that raises the hypothesis of a putative functional connection of both events in EMT activation and metastatic spreading [38, 39]. Similar to what we have previously reported concerning the role of *BRAF* and *RAS* mutations in the spread and homing of mutated *BRAF* or *RAS* cells in lymph nodes [40, 41], in this study, we also saw that *TERT* amplification disseminated with PTC cells, but did not drive the development of LNMs in PTCs. Metastatic tumor expansion into lymph nodes can occur independently of *TERT* amplification, a finding consistent with the lack of correlation of *TERT* amplification with tumor multifocality, which is known to increase the likelihood of developing LNMs in PTCs. Importantly, the concurrence of other oncogenic drivers (*TPM*, *BRAF*, or *RAS*) and *TERT* amplification does not improve the correlations of prognostic relevance observed with *TERT* amplification per se. On the contrary, some of the observed correlations become just a tendency towards an association or worsened notably. Only the conjunction of *TERT* amplification and *TPM* determined that the tendency towards the association observed between *TERT* amplification and age became statistically significant.

Our data clearly point to a greater impact of *TERT* amplification than *TPM* on the prognosis of PTCs. The only prognostic traits with which *TPM* were significantly correlated were the patient’s age, tumor stage, and DOD status. In contrast to what was observed with *TERT* amplification, the concurrence of *TPM* with *TERT* amplification or *RAS* mutations results in the appearance of statistically significant associations with clinical-pathological traits of poor prognosis (vascular invasion, DMs at diagnosis and/or during follow-up, and metachronous DMs) that with *TPM* per se were not significant. The latter does not apply to the coexistence of *TPM* and *BRAF* mutations, which even leads to the loss of the correlation found between *TPM* and DOD status.

According to what we and others have previously reported, *RAS* mutations also have a greater impact on the prognosis of patients with PTC than *BRAF* mutations [40]. While *BRAF* mutations were found to correlate only with the presence of focal tall cell futures and the development of tumor recurrences, *RAS* mutations, as formerly shown [40], demonstrated to be significantly associated to age, DMs at diagnosis and/or during the follow-up, metachronous DMs, developed during the follow-up, and DOD patient status.

See “online resource 4” for discussion of impact of *BRAF* mutations in recurrence-free survival and risk of death of disease in PTC patients.

We demonstrate for the first time that *TERT* amplification is associated with a lower probability of recurrence-free survival and a greater risk of death of disease in PTC patients, findings that are consistent with the statistically significant correlation found between *TERT* amplification and the development of metachronous DMs, during patient follow-up. A relationship with tumor recurrence and shorter survival that has been previously reported in different tumor types [3, 42–44]. In our study, we show that *TERT* amplification is a better predictor of tumor relapse than *TPM*, extrathyroidal extension, tumor multifocality or tumor size ≥ 5 cm, DMs at diagnosis, and stage at diagnosis I–II vs. III–IV. Furthermore, *TERT* amplification predicts tumor relapse independently of other variables that also exhibit predictive value such as the presence of foci of infiltrative insulae of tumor cells, surrounded by a desmoplastic reaction at the advancing edge of the tumor, presence of areas of focal tall cell appearance, age ≥ 55 years old, male sex, vascular invasion, LNMs at diagnosis, and *BRAF* mutations. Likewise, our findings also evidence that *TERT* amplification is a better predictor of tumor-related death than *TPM*, male sex, tumor multifocality or tumor size ≥ 5 cm, vascular invasion, DMs at diagnosis and/or follow-up, and stage at diagnosis I–II vs. III–IV. Additionally, we show that *TERT* amplification is able to prognosticate lower survival independently of other variables that also display predictive value such as age ≥ 55 years old, extrathyroidal extension, and *BRAF* mutations. In contrast to *TERT* amplification, the presence of *BRAF* mutations increases the likelihood of recurrence-free probability and decreases the risk of tumor-related death. It has a protective effect.

Beyond the two most common *TPM*, which selectively recruit the ETS transcription factor GABP to activate *TERT*, the mechanism of telomerase reactivation in case of *TERT* amplification in thyroid cancer is poorly characterized. Mechanistic studies have been approached in bladder and glioblastomas cell lines bearing constructs that mimic *TERT* duplications containing a de novo native ETS motif for the GABP transcription factor complex. ETS-harboring *TERT* duplications were first reported in PTC and PDC [7] and shortly after in glioblastomas [45] and other tumors (glioblastoma multiforme, bladder urothelial carcinoma, hepatocellular carcinoma, squamous cell carcinoma of the oropharynx, duodenal adenocarcinoma, and malignant phyllodes breast tumor) [46]. Barger et al. have shown that the mechanism underlying *TERT* reactivation in glioblastomas and urothelial bladder carcinomas is analogous to that observed in the case of *TPM*. *TERT* activation requires the additional ETS binding site provided by a wild-type sequence duplication and also of GABP transcription factor complex [46]. The GABPB1L containing GABP complex binds to sequences with duplicated native ETS sites and increases *TERT* promoter activity [46]. The knockdown of GABPA by means of siRNAs revealed a dramatic reduction of TERT expression [46]. Similar studies in thyroid cancer cell lines are needed to determine if the GABP tetramer is also an essential regulator of *TERT* amplification, mimicking *TPM* in activation strength and mechanism.

## Concluding Remarks

*TERT* amplification is independently associated with PTC-related recurrence and death.

Our findings indicate that PTCs can be stratified into clinically prognostic relevant categories based on the presence or not of *TERT* amplification in the cells. *TERT* amplification status might prove an objective and useful adjunct to current classifications systems in predicting PTC recurrence and survival.

The detection of discrete subclonal *TERT* activation events (*TERT* amplification and/or *TPM*) in specific histologic parts of the tumors may be useful early information regarding possible escape or resistance to targeted therapies.

The PTCs with DMs can be considered the first stage in thyroid tumor progression and dedifferentiation from which an accumulation of *TERT* activating events is prone to occur.

Since a variety of mechanisms are involved in TERT re-expression and telomerase activation, testing only of *TPM* has a limited prognostic value in advanced thyroid cancers. To determine the true utility of *TPM* in a clinical setting and know how to best target TERT regulation is mandatory to further understand how thyroid cancer cells that do not harbor *TPM* activate *TERT* through various regulatory processes. Major gaps in our understanding of telomerase regulation remain to be elucidated. A better understanding of the mechanisms responsible of TERT upregulation may significantly impact on the rationale of future treatment modalities for patients with aggressive PTCs.

A key challenge is how to translate these findings into a telomerase-based therapeutic strategy. Given the high prevalence of *TERT* alterations in different cancer types, there have been ongoing efforts to target components of the telomerase holoenzyme. Although such approaches are not currently included in clinical use, they represent exciting future cancer therapy strategies.

## Supplementary Information

Below is the link to the electronic supplementary material.

Supplementary Figure 1 (PNG 174 KB)

Supplementary file1 Impact of *TPM* and *BRAF* mutations on disease-related recurrence in PTC patients with LNMs but without DMs. Kaplan-Meier estimate of recurrence-free probability in PTC patients with information available regarding the exact moment of tumor relapse. Patients were dichotomized according to: (**a**) the presence of TPM; (**b**) the presence of BRAF mutations; (**c**) the concurrence of *TPM* and *BRAF* mutations (TIF 200 KB)

Supplementary Figure 2 (PNG 162 KB)

Supplementary file2 Impact of *TPM* and *BRAF* mutations on disease-related survival in PTC patients with LNMs but without DMs. Kaplan-Meier estimate of likelihood of disease-related death in PTC patients with information available regarding the exact moment of DOD. Patients were dichotomized according to: (**a**) the presence of TPM; (**b**) the presence of BRAF mutations; (**c**) the concurrence of *TPM* and *BRAF* mutations (TIF 190 KB)

Supplementary file3 (PDF 159 KB)

Supplementary file4 (PDF 139 KB)

Supplementary file5 (PDF 843 KB)

Supplementary file6 (PDF 164 KB)

## Data Availability

No datasets were generated or analysed during the current study.
