# Correction to: TERT Amplification a Risk Stratification Marker in Papillary Thyroid Carcinoma, Significantly Correlated with Tumor Recurrence and Survival

**DOI:** 10.1007/s12022-025-09881-0

**Published:** 2025-10-30

**Authors:** Sara Gil-Bernabé, Noa Feás-Rodríguez, Enrique Pérez-Riesgo, Miriam Corraliza-Gómez, Joaquín Fra Rodríguez, Ginesa García-Rostán

**Affiliations:** 1 Institute of Biomedicine and Molecular Genetics (IBGM), Valladolid University, Valladolid, Spain; 2 School of Medicine, Valladolid University, Valladolid, Spain; 3 European Miguel de Cervantes University, Valladolid, Spain; 4 University of Cádiz, Cádiz, Spain; 5 Hospital Río Hortega, Valladolid, Spain

**Correction to: Endocrine Pathology (2025) 36:15**

10.1007/s12022-025-09853-4

In the original version of this article, the given and family names of all authors were incorrectly structured. See details below.

**Table:** 

**Incorrect presentation of author names in the original publication**	**Correct presentation of author names**
Gil-Bernabé Sara	Sara Gil-Bernabé
Feás-Rodríguez Noa	Noa Feás-Rodríguez
Pérez-Riesgo Enrique	Enrique Pérez-Riesgo
Corraliza-Gómez Miriam	Miriam Corraliza-Gómez
Fra Rodríguez Joaquín	Joaquín Fra Rodríguez
García-Rostán Ginesa	Ginesa García-Rostán

The original article has been corrected.

