# Supplementary figures and images for: TERT Amplification a Risk Stratification Marker in Papillary Thyroid Carcinoma, Significantly Correlated with Tumor Recurrence and Survival
Source: Endocr Pathol. 2025 Apr 24;36(1):15. doi: 10.1007/s12022-025-09853-4 (PMC12021720; doi:10.1007/s12022-025-09853-4)

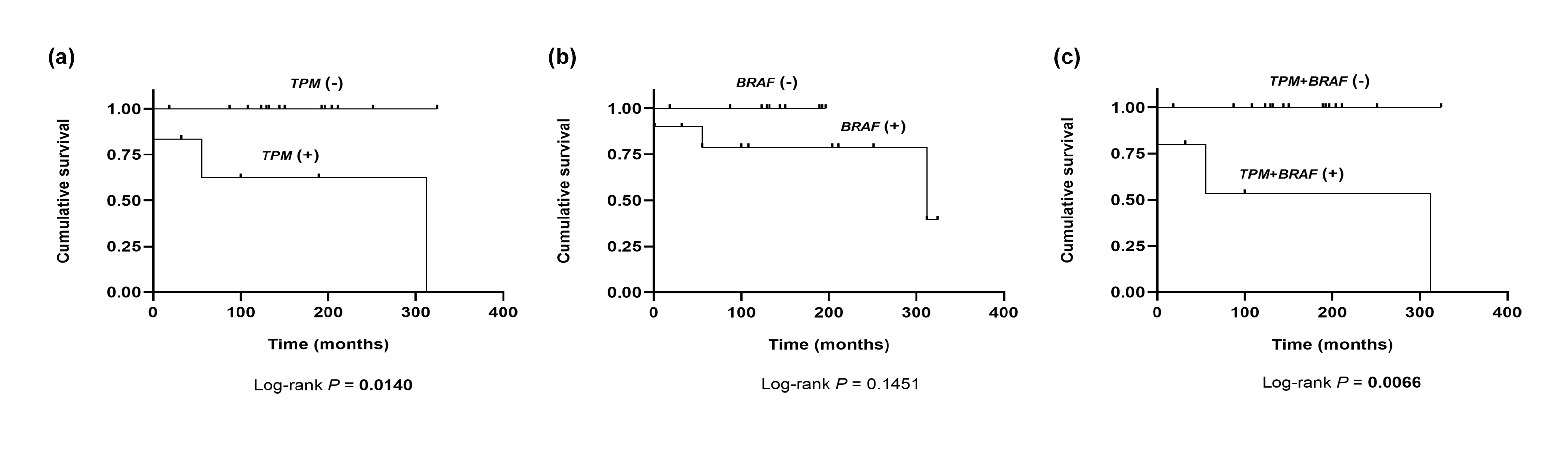

Supplement: Supplementary file 1 — (PNG 174 KB) [file 12022_2025_9853_Fig6_ESM.png]

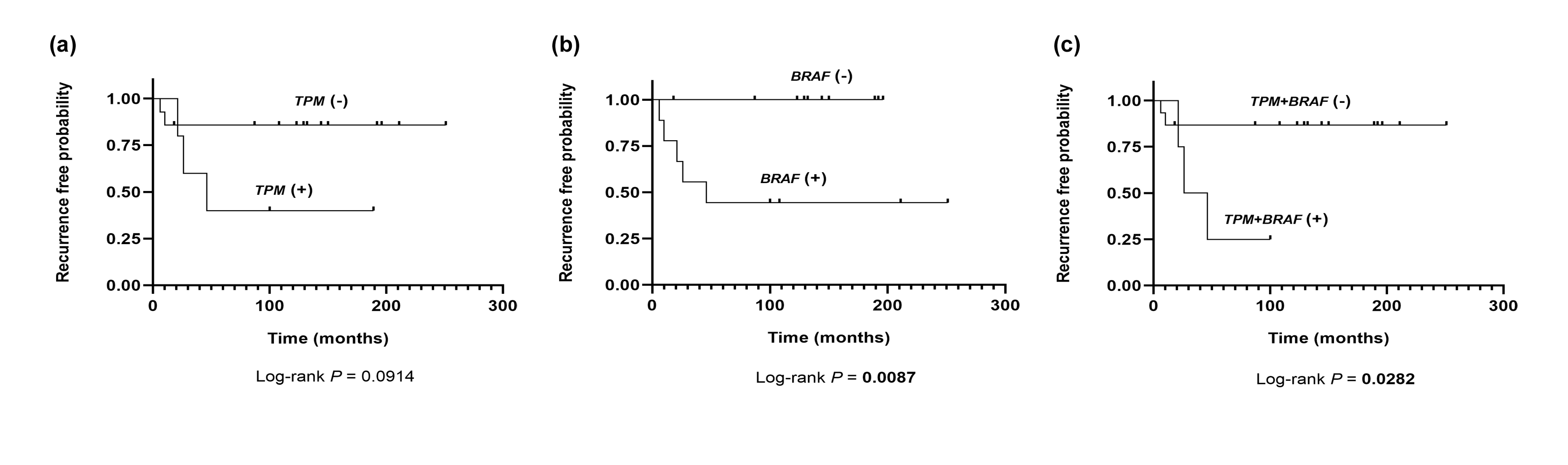

Supplement: Supplementary file 2 — Supplementary file1 Impact of TPM and BRAF mutations on disease-related recurrence in PTC patients with LNMs but without DMs. Kaplan-Meier estimate of recurrence-free probability in PTC patients with information available regarding the exact moment of tumor relapse. Patients were dichotomized according to: (a) the presence of TPM; (b) the presence of BRAF mutations; (c) the concurrence of TPM and BRAF mutations (TIF 200 KB) [file 12022_2025_9853_MOESM1_ESM.tif]

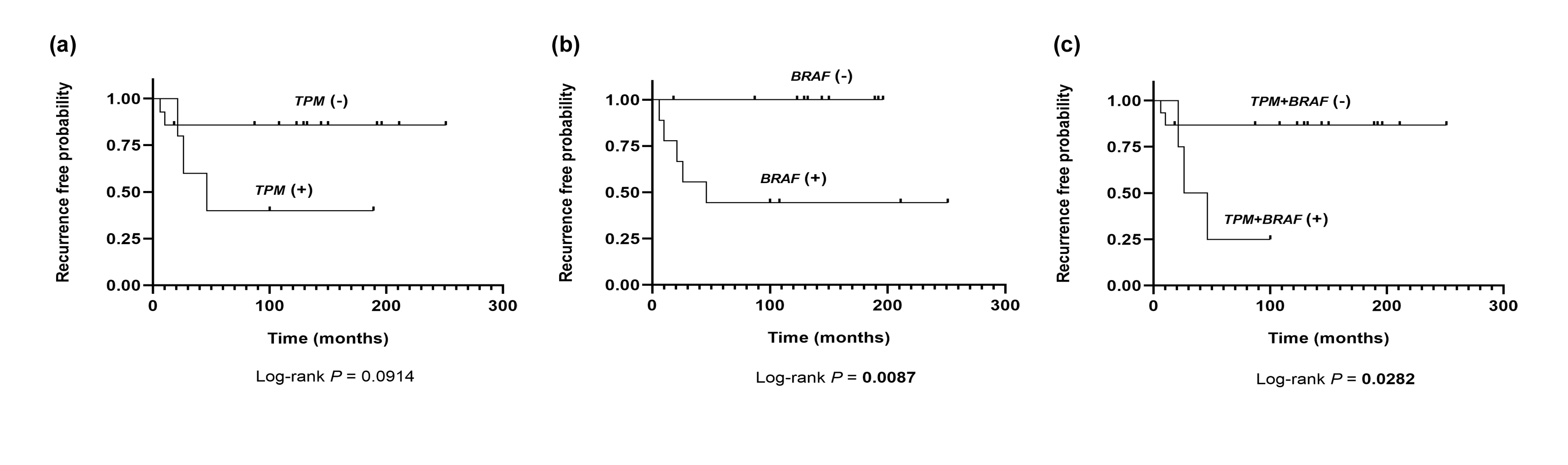

Supplement: Supplementary file 3 — (PNG 162 KB) [file 12022_2025_9853_Fig7_ESM.png]

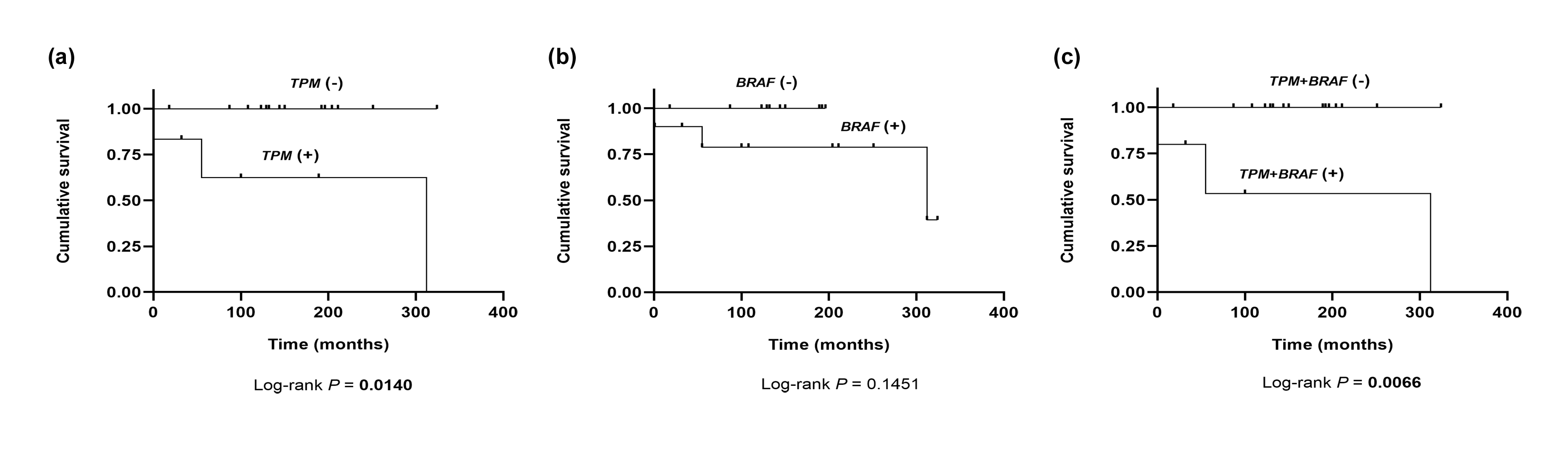

Supplement: Supplementary file 4 — Supplementary file2 Impact of TPM and BRAF mutations on disease-related survival in PTC patients with LNMs but without DMs. Kaplan-Meier estimate of likelihood of disease-related death in PTC patients with information available regarding the exact moment of DOD. Patients were dichotomized according to: (a) the presence of TPM; (b) the presence of BRAF mutations; (c) the concurrence of TPM and BRAF mutations (TIF 190 KB) [file 12022_2025_9853_MOESM2_ESM.tif]
