# Supplementary material for: TERT Amplification a Risk Stratification Marker in Papillary Thyroid Carcinoma, Significantly Correlated with Tumor Recurrence and Survival
Source: Endocr Pathol. 2025 Apr 24;36(1):15. doi: 10.1007/s12022-025-09853-4 (PMC12021720; doi:10.1007/s12022-025-09853-4)
Supplement: Supplementary file 5 — Supplementary file3 (PDF 159 KB) [file 12022_2025_9853_MOESM3_ESM.pdf]

Online resource 1 paper *Endocrine Pathology* DOI: 10.1007/s12022-025-09853-4

METHODS:

DNA isolation

Hematoxilyn-eosin stained sections were microscopically reviewed to determine the percentage of tumor cells comprising each of the areas to be genotyped, and to demarcate the boundaries of the pT areas microdissected within the same FFPE tissue section or different tissue sections from different tumor blocks. Whenever necessary, samples were manually microdissected to eliminate areas of non-tumoral tissue (normal thyroid follicles in the vicinity of pTs, non-tumoral lung parenchyma encircling lung DMs, osseous spikes /trabeculae or soft tissues surrounding bone DMs,…..) and achieve at least 90-95% of tumor purity. The amount of tissue cut varied in function of the size and cellularity of each of the areas to be genotyped. After deparaffination with Xylene at 65ºC and hydration with ethanol, all of the microdissected tissues areas were processed with the commercial kit “GeneJET FFPE DNA purification kit” (Thermo Fisher Scientific, Waltham, MA) to extract genomic DNA. The number of tumor sampled areas that were genotyped per case ranged between 2 and 10.

Analysis of CNV at *TERT*

A multiplex ligation-dependent probe amplification (MLPA) method was used to investigate changes in *TERT* gene dosage (5p15.33) [1]. A targeted probe mixture specifically designed to finely map different *TERT* gene exons was included in a multiplex PCR reaction. Several reference probes, which hybridized at autosomal chromosomal locations that are relatively silent or stable [*FAF1*, *CACNA1S*, *DYSF*, *ACVR2A*, *ATP8A1*, *PKHD1*, *PCSK5*, *STXBP1*, *UPF2*, *SMPD1*, *SPG11*], were added to the probe mix for data normalization. In each MLPA experiment were also included several reference non-tumoral /normal single gene copy number DNAs, which were extracted with the same DNA extraction kit as the tumoral samples, and derived from the same source of tissue (FFPE normal thyroid tumor blocks).

Each MLPA probe included in the probe mix consist of two fluorescently labeled (FAM) hemiprobes or oligonucleotide sequences [50-60 bp and 60-450 bp], which hybridize only with the target gene sequence [nucleotide sequence in which we are interested in], adjacent to each other so that we can join / glue them together with a thermostable ligase. Once hybridization and ligation have occurred, we proceed to the exponential amplification of all the probes present in our mix using a multiplex PCR. A universal pair of primers, which recognizes complementary nucleotide sequences present at the 5' and 3' ends of each probe, is used in the PCR amplification step. The labeled hemiprobes/oligonucleotides that do not bind to the target sequence, as they only contain one of the primers, cannot be amplified during the PCR and, therefore, do not generate any fluorescent signal. In order to differentiate and separate by capillary electrophoresis each of the probes amplified in the multiplex-PCR, a filler nucleotide sequence (stuffer sequence), of different length for each of the probes present in the mixture, is added to one of the hemiprobes. The MLPA reaction itself consists of 4 steps: (1) Denaturation of 20-200ng DNA/opening of the double helix, incubation with the probes for 16 hours, and hybridization of the probes; (2) Ligation of hemiprobes; (3) Ligase inactivation and amplification by multiplex PCR; and (4) Separation of PCR products by capillary electrophoresis – Fragment analysis.

All 215 tumoral sampled areas were analyzed at least in duplicate. The amplification products were sorted and quantified by capillary electrophoresis using an automatic sequencer, with a ROX-labelled internal size standard. The Coffalyser tool, developed by MRC Holland, was used for the copy number variation analyses. Intra-sample normalization and inter-sample comparison was performed in all cases. A probe ratio between 0.8 and 1.2 was considered normal gene copy number, a ratio of 0 was considered as homozygous deletion, a ratio between 0.1 and 0.7 was defined as heterozygous deletion, a ratio between 1.30 and 1.55 was

classified as borderline increase in gene copy number / heterozygous duplication, a ratio between 1.60 and 1.80 was cataloged as moderate increase in gene copy number / heterozygous duplication, a ratio between 1.85 and ≥ 2 was considered as homozygous duplication / heterozygous triplication / gene amplification

Mutational Analysis

Genomic DNA was evaluated for the existence of mutations at 6 of the most idiosyncratic driver oncogenes associated with thyroid cancer progression, dedifferentiation and aggressiveness (*BRAF*, *HRAS*, *NRAS*, *KRAS*, *PIK3CA*, *TERTp*), including 5 of the 7 "*significantly mutated genes*" plus the *TERT* promoter reported in PTCs by the Cancer Genome Atlas Research Network [2].

Mutations at the activation loop of the *BRAF* gene (exon 15), the GTP-binding domain and the GTP-ase domain of the three members of the *RAS* gene family (*H-*, *N-*, and *K-RAS*) were investigated by means of PCR-SSCP-sequencing of mobility shifts, following our previously described protocols [3,4]. Mutations at *PIK3CA* and *TERTp* were characterized by PCR followed by direct sequencing. In the case of the *PIK3CA* gene, were used the primers and PCR conditions that we have previously described [5]. The primers and PCR conditions for *TERTp* were devised in the laboratory, and are available upon request. All of the detected mutations were further verified by at least one independent analysis from a new DNA template.

Statistical Analyses

SPSS software (SPSS Inc, Chicago, IL, USA) was chosen for statistical analyses. The two-tailed Fisher´s exact test and the χ^2 test were applied to assess the putative associations between the altered genotypes and the clinical-pathological features (see Table-1) recorded for each patient. *P* values ≤ 0.05 were considered statistically significant. A trend of correlation was assumed when the *P* values ranged between 0.05 and 0.18.

The likelihood of tumor-related recurrence was investigated in 34 patients [19 PTCs without DMs and 15 PTCs with DMs] with a follow-up period long enough to develop a true tumor recurrence. Seven cases were excluded, 2 of which were alive with disease (AWD) patients in whom we did not know the exact time of tumor recurrence, 4 were patients who died due to the tumor between 1 and 7 months after diagnosis and, thus, there was no material time to develop a true tumor recurrence, and 1 was a patient with NED, with a very short follow-up, with no possibility of obtaining additional information on his clinical evolution.

Disease-specific survival was evaluated in 40 cases [20 patients without DM and 20 patients with DM). A patient without DM was excluded from the analyses due to have a short follow-up, and being impossible to obtain additional information.

Disease-specific survival and recurrence-free probability were assessed by Kaplan-Meier plots and the Log-rank test. Patients who died as a result of the tumor (death of disease, DOD) were classified as uncensored, whereas those who were AWD or alive without disease (no evidence of disease, NED) were coded as censored. Most of the cases analyzed without DM (86%) had NED after a mean follow-up of 144 months (12 years).

Univariate analysis was performed in PTC patients to estimate the relative risk (RR) of disease-related recurrence or death associated with clinical-pathological parameters or altered genotype. Multivariate analysis was performed to assess whether or not the presence of *TERT* amplification in PTCs was an independent predictor of tumor recurrence or patient survival. The selection of variables results crucial to identify those variables that are significantly associated with the time to an event (recurrence o survival) in Cox proportional hazards regression models. In order to find the best and simplest multivariate model, which best explains the dependent variable time elapsed until an event (recurrence or survival), a top-down approach was followed. To do this, first a complete model is built. A model, which contains all the possible predictor / explanatory / independent variables that, both in the series under study and in the published literature, have been shown that may significantly impact on PTC relapse and/or death. Next, from this complete model a narrow down of variables is performed, eliminating those that do not have predictive

capacity. This filtering is carried out both in forward and backward directions following the Akaike Information Criterion (AIC). The AIC evaluates, on the one hand, whether the model improves by introducing or eliminating a possible predictor variable and, on the other hand, penalizes the introduction of a new variable since always, even if a variable does not have predictive capacity, the error of the model decreases. Thus, if the improvement of the model by introducing a certain predictor variable exceeds the penalty of introducing one more predictor variable, then such variable really has predictive capacity and, consequently, is capable of predicting and/or explaining the dependent variable (recurrence or survival). In this way the most parsimonious multivariate model is selected, balancing model complexity and goodness of fit. The final selected multivariate model includes all those variables, among all possible explanatory variables initially considered, that contribute the most to explain "time to an event" (recurrence or survival), which implies their relevance to survival or event risk. The AIC-based selection focuses on overall model performance rather than in individual hypothesis tests. Notably, this approach is robust for small sample sizes. Sometimes, a variable selected through AIC may not appear statistically significant (e.g., p-value > 0.05), however, it might still be important to include it in the model because it contributes to the overall predictive performance. AIC serves as a complementary method to traditional hypothesis testing, focusing on the variable's contribution to the model's predictive ability rather than its individual statistical significance. The Wald test was done to find out if explanatory variables in the different prognostic models were significant. The likelihood ratio test was performed to assess the global significance of the prognostic model.

REFERENCES:

1. Schouten JP, McElgunn CJ, Waaijer R, Zwijnenburg D, Diepvens F, Pals G. (2002) Relative quantification of 40 nucleic acid sequences by multiplex ligation-dependent probe amplification. Nucleic Acids Res, 30(12):e57. DOI: 10.1093/nar/gnf056
2. Cancer Genome Atlas Research Network. (2014) Integrated genomic characterization of papillary thyroid carcinoma. Cell, 159(3):676-690. DOI: 10.1016/j.cell.2014.09.050
3. Costa AM, Herrero A, Fresno MF, Heymann J, Alvarez JA, Cameselle-Teijeiro J, García-Rostán G. (2008) BRAF mutation associated with other genetic events identifies a subset of aggressive papillary thyroid carcinoma. Clin Endocrinol (Oxf), 68:618-634. DOI: 10.1111/j.1365-2265.2007.03077.x
4. Garcia-Rostan G, Zhao H, Camp RL, Pollan M, Herrero A, Pardo J, Wu R, Carcangiu ML, Costa J, Tallini G. (2003) Ras mutations are associated with aggressive tumor phenotypes and poor prognosis in thyroid cancer. J Clin Oncl, 21:3226-3235. DOI: 10.1200/JCO.2003.10.130
5. García-Rostán G, Costa AM, Pereira-Castro I, Salvatore G, Hernandez R, Hermsem MJ, Herrero A, Fusco A, Cameselle-Teijeiro J, Santoro M. (2005) Mutation of the PIK3CA gene in anaplastic thyroid cancer. Cancer Res, 65:10199-10207. DOI: 10.1158/0008-5472.CAN-04-4259
