# Supplementary material for: TERT Amplification a Risk Stratification Marker in Papillary Thyroid Carcinoma, Significantly Correlated with Tumor Recurrence and Survival
Source: Endocr Pathol. 2025 Apr 24;36(1):15. doi: 10.1007/s12022-025-09853-4 (PMC12021720; doi:10.1007/s12022-025-09853-4)
Supplement: Supplementary file 6 — Supplementary file4 (PDF 139 KB) [file 12022_2025_9853_MOESM4_ESM.pdf]

Online resource 2 paper *Endocrine Pathology* DOI: 10.1007/s12022-025-09853-4

Activation in PTCs of other oncogenic drivers in thyroid carcinogenesis: *BRAF*, *RAS* [*H-*, *K-*, and *N-RAS*], and *PIK3CA*. Clonality and spread with metastatic PTC cells

- *BRAF mutations, clonality and spread with metastatic PTC cells* – **See fig1**

A 54% of the PTCs were mutated at *BRAF* [11 of 21 cases without DMs (52%) and 11 of 20 cases with DMs (55%)]. The most common mutation was the V600E, found in 82% of the mutated cases [all the mutated cases without DMs and 64% of the cases with DMs]. Among the series of patients with DM were also found other mutations reported at COSMIC such as K601 (9% mutated cases), Q609* (18% mutated cases), T589I (9% mutated cases), T599I (9% mutated cases), and Q612* (9% mutated cases). Two PTC cases with DMs showed double *BRAF* mutants, a finding more frequently observed among more aggressive, less differentiated thyroid tumors.

When considering all the tumor samples analyzed in each PTC mutated at *BRAF*, regardless of the histotype observed on each area of the case under investigation and whether the sample genotyped corresponded to a pT, LNM or DM, 71% of the *BRAF* mutations were found to be clonal, were present in all the analyzed areas [91% of the cases without DMs and 50% of the cases with DMs]. In 75% of the mutated PTCs with more than one area of pT analyzed, the mutation was clonal. Mostly PTCs from the series of patients with DMs (4/6-67%) [Fig1]. In 100% of the mutated PTCs with more than one area of LNM analyzed, the mutation was clonal, was present in all the different LNMs screened [Fig1]. In 50% of the mutated PTCs with more than one area of DM analyzed, the mutation was clonal, was present in all the different synchronous and/or metachronous DMs studied [Fig1].

In 86% of the PTCs mutated at *BRAF* the mutation migrated with metastatic PTC cells to LNMs and/or DMs. In 3 cases *BRAF* was circumscribed to the pT [Fig1].

- *RAS* [*H-*, *K-*, and *N-RAS*] *mutations, clonality and spread with metastatic PTC cells* – **See fig1**

A 24% of the PTCs were mutated at *RAS* [1 of 21 cases without DMs (5%) and 9 of 20 cases with DMs (45%)]. Most of the PTCs mutated in *RAS* were PTCs with DMs (90%). Of the 3 *RAS* genes, *K-RAS* was activated in 6 cases (60%), *N-RAS* in 3 cases (30%), and *H-RAS* in 2 cases (20%). All 6 cases mutated at *K-RAS* displayed a G12D mutation, and 2 cases harboured an additional G13S mutation, which seemingly developed "*de novo*" at LNMs. Two of the cases mutated at *N-RAS* had the Q61R mutation, which apparently emerged "*de novo*" at the DMs, and the third case had the Q61K mutation, which was present in all the genotyped areas of pT and DMs. The two cases mutated at *H-RAS* showed the Q61R mutation, which in one of them was restricted to the pT. Three of the mutated cases (30%), preferentially PTCs with DMs (67%), exhibited double *RAS* mutations, a finding more common among less differentiated thyroid tumors.

When considering all the tumor samples analyzed in each PTC mutated at *RAS*, regardless of the histotype observed on each area of the case under investigation and whether the sample genotyped corresponded to a pT, LNM or DM, 56% of the *RAS* mutations were found to be clonal, were present in all the analyzed areas [1 case without DMs and 4 cases with DMs]. In 75% of the mutated PTCs with more than one area of pT analyzed, the mutation was clonal [Fig1]. In 100% of the mutated PTCs with more than one area of LNM analyzed, the mutation was clonal, was present in all the different LNMs screened [Fig1]. In 100% of the mutated PTCs with more than one area of DM analyzed, the mutation was clonal, was seen in all the different synchronous and/or metachronous DMs studied [Fig1].

In 67% of the PTCs mutated at *RAS* the mutation migrated with metastatic PTC cells to LNMs and/or DMs. In the other 3 cases the mutation seemingly originated *"de novo"* at the metastatic niche [Fig1]. Two of them showed the Q61R mutation at *N-RAS* and one the double mutant G12D plus G13S at *K-RAS*.

- *PIK3CA mutations, clonality and spread with metastatic PTC cells* – **See fig1**

A 7% of the PTCs were mutated at *PIK3CA* [1 of 21 cases without DMs (5%) and 2 of 20 cases with DMs (10%)]. Most of the PTCs mutated in *PIK3CA* were PTCs with DMs (67%). Two cases displayed the mutation E545K and 1 case the mutation M1043I. Although both mutations migrated with metastatic PTC cells to LNMs and DMs, neither one was clonal at the pT, they were not seen in all the characterized areas of pT. The mutation M1043I was also not present in all the DMs analyzed in the case.

Activation in PDCs and ATCs of other oncogenic drivers in thyroid carcinogenesis: *BRAF*, *RAS* [*H-*, *K-,* and *N-RAS*], and *PIK3CA*. Clonality, spread of mutations with tumor dedifferentiation within pT and molecular heterogeneity

- *Poorly Differentiated Carcinomas* – **See fig2**:

A 20% of the PDCs investigated were found to be mutated at *BRAF* [2 cases V600E and 1 case Q612*]. In 2 of the 3 mutated cases, concurrent better differentiated areas within the pT were analyzed and the mutation was found to move from the better differentiated areas to the PDC areas. It was a clonal event, present in all the areas genotyped.

RAS mutations were present in 33% of the PDCs analyzed. The most frequently mutated genes were *K-RAS* and *N-RAS*, both present in 80% of the mutated PDCs. A 60% of the mutated PDCs showed double mutations, with concurrent mutations in *N-RAS* (Q61K) and *K-RAS* (G12S or G12D). In 3 of the mutated cases (60%) was analyzed more than one area within the pT, including better differentiated areas [FV-PTC or Tall PTC], and the mutation was clonal, was present in all the areas characterized, progressing from better differentiated areas to poorly differentiated areas.

Only one PDC was found mutated at *PIK3CA* (7%) and the mutation was restricted to the PDC area, being the well-differentiated area WT.

Tumor genetic heterogeneity was demonstrated in 60% (6/10) of the mutated PDCs. The two most frequently activated genes in cases with heterogeneity were *RAS* (83%) and *TERT* [83% - 4 cases by TPM and 1 case by amplification]. The third one was *BRAF* (50%). Two genes were concurrently activated in 33% of the mutated PDCs with genetic heterogeneity. Three genes coexisted in 67% of the mutated PDCs with genetic heterogeneity.

- *Anaplastic Thyroid Carcinomas* – **See fig2**:

A 37% of the ATCs were mutated at *BRAF*. The most common type of mutation was V600E (69%), followed by Q612* (15%), G615E (8%), and T599I (8%). In 50% of the mutated cases with more than one area of pT analyzed, the mutation was found to be clonal. One of the latter 2 cases included a concurrent better differentiated area and the mutation progressed from the better differentiated area to the ATC area. In the other 2 mutated cases, in which a concurrent better differentiated area within the pT was analyzed, the *BRAF* mutation either emerged *"de novo"* in the ATC areas, or it was circumscribed to the best differentiated area. In one of the 13 mutated ATCs was characterized a LNM and the mutation spread with metastatic cells to the LNM.

A 26% of the ATCs were mutated at *RAS*. The most common type of mutation was the Q61R at *N-RAS* (6/9 – 67%), followed by the G12D at *K-RAS* (3/9 – 33%). In 3 of the mutated ATCs were genotyped different areas within the pT, including better differentiated areas, and the analysis showed that mutations progressed from

the better differentiated areas to the ATC areas in all 3 cases (100%), but were clonal, present in all areas of the pT, in only 2 cases (67%). In one of the 9 mutated ATCs was characterized a LNM and the mutation spread with metastatic cells to the LNM.

PIK3CA mutations were found in 26% of the ATCs. All of them at de kinase domain (exon 20). In none of the mutated cases was genotyped more than one area within the pT.

Among ATCs, 66% (19/29) of the mutated cases displayed molecular heterogeneity. *TERT* gene was activated in all 19 cases [74% by TPM, 11% by amplification and 16% by both events]. The second most frequently concurrently activated gene was *BRAF* (68%). Both *RAS* and *PIK3CA* were activated in 42% of the cases. *N-RAS* and *K-RAS* were activated in 75% and 25% of the ATCs with molecular heterogeneity that were mutated at *RAS*. Two genes were concurrently activated in 58% of the mutated ATCs with genetic heterogeneity. Three genes coexisted in 32% of the mutated ATCs with genetic heterogeneity. Four genes coexisted in 11% of the mutated ATCs with genetic heterogeneity.
