# Supplementary material for: TERT Amplification a Risk Stratification Marker in Papillary Thyroid Carcinoma, Significantly Correlated with Tumor Recurrence and Survival
Source: Endocr Pathol. 2025 Apr 24;36(1):15. doi: 10.1007/s12022-025-09853-4 (PMC12021720; doi:10.1007/s12022-025-09853-4)
Supplement: Supplementary file 7 — Supplementary file5 (PDF 843 KB) [file 12022_2025_9853_MOESM5_ESM.pdf]

"Online resource 3" paper *Endocrine Pathology* DOI: 10.1007/s12022-025-09853-4

Evaluation, in different tumor types, of TERT mRNA expression using the web resource cBioPortal (http://cbioportal.org) for exploring, visualizing, and analyzing multidimensional cancer genomics data sets.

Genome data sets of different types of cancers, including information on TERT mRNA expression and copy number alterations, were uploaded to cBioPortal to examine the level of TERT expression in cases with *TERT* amplification and cases with *TERT* copy number gains. All the different analyses approached showed that *TERT* amplification was associated with much higher expression of TERT than *TERT* copy number gains.

TCGA – LUNG SQUAMOUS CELL CARCINOMA [Hoadley KA, Yau C, Hinoue T, Wolf DM, Lazar AJ, Drill E, Shen R, Taylor AM, Cherniack AD, Thorsson V, Akbani R, Bowlby R, Wong CK, Wiznerowicz M, Sanchez-Vega F, Robertson AG, Schneider BG, Lawrence MS, Noushmehr H, Malta TM; Cancer Genome Atlas Network; Stuart JM, Benz CC, Laird PW. (2018) Cell-of-Origin Patterns Dominate the Molecular Classification of 10,000 Tumors from 33 Types of Cancer. Cell, 173(2): 291–304.e6. https://doi.org/10.1016/j.cell.2018.03.022]

498 samples

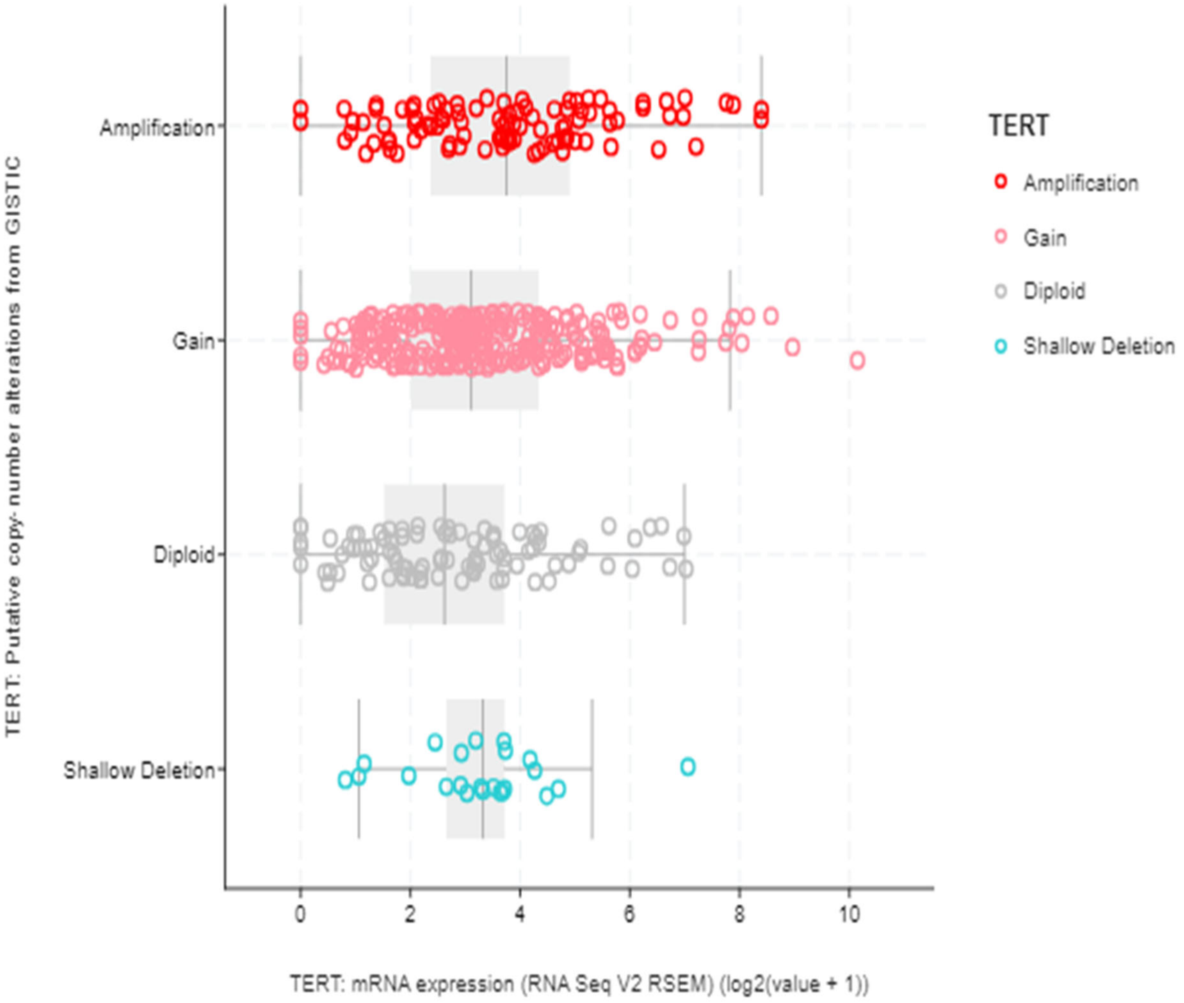

TCGA – BLADDER UROTHELIAL CARCINOMA [Hoadley KA, Yau C, Hinoue T, Wolf DM, Lazar AJ, Drill E, Shen R, Taylor AM, Cherniack AD, Thorsson V, Akbani R, Bowlby R, Wong CK, Wiznerowicz M, Sanchez-Vega F, Robertson AG, Schneider BG, Lawrence MS, Noushmehr H, Malta TM; Cancer Genome Atlas Network; Stuart JM, Benz CC, Laird PW. (2018) Cell-of-Origin Patterns Dominate the Molecular Classification of 10,000 Tumors from 33 Types of Cancer. Cell, 173(2): 291–304.e6. https://doi.org/10.1016/j.cell.2018.03.022]

402 samples

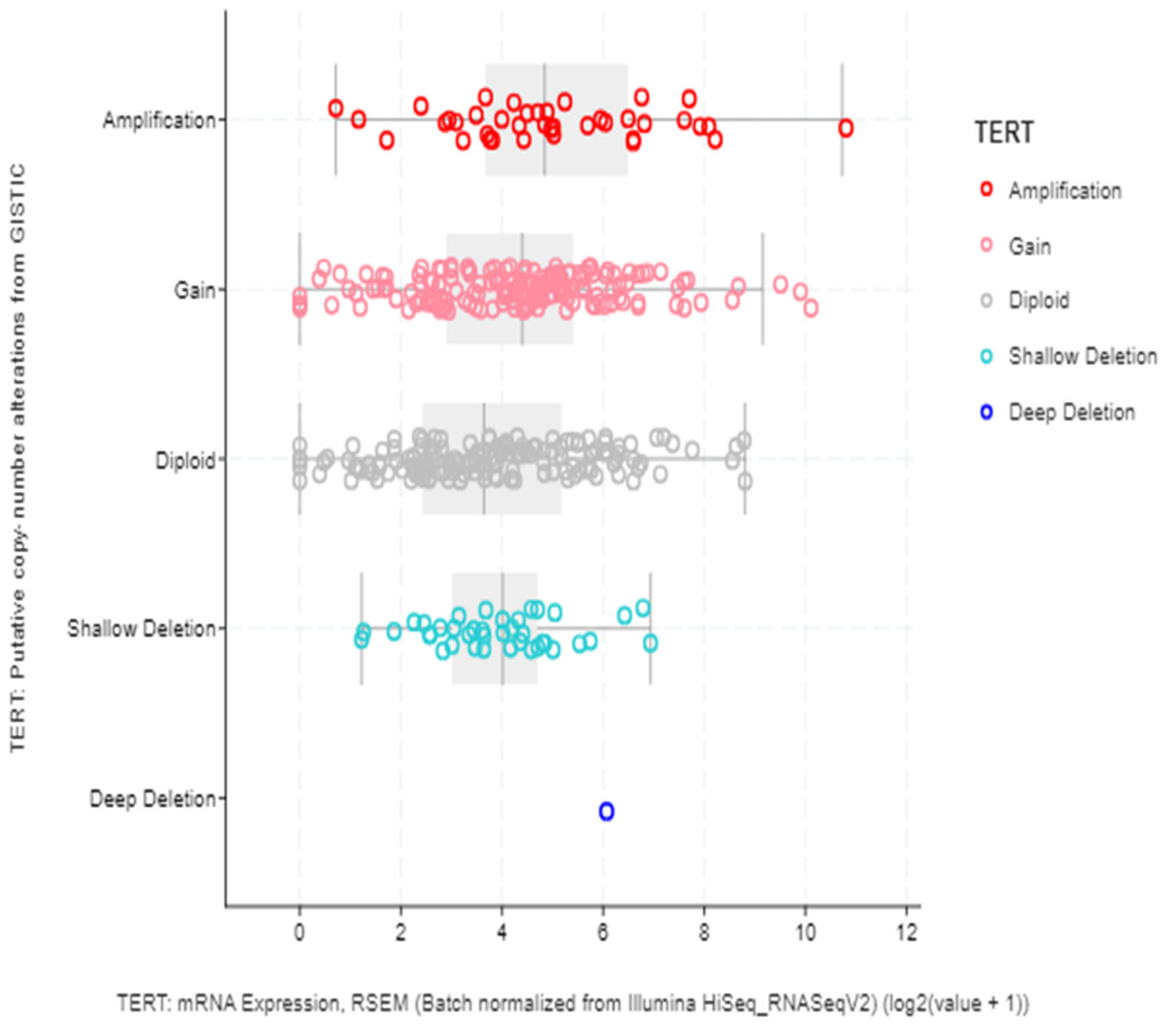

TCGA – METASTATIC MELANOMA [Van Allen EM, Miao D, Schilling B, Shukla SA, Blank C, Zimmer L, Sucker A, Hillen U, Foppen MHG, Goldinger SM, Utikal J, Hassel JC, Weide B, Kaehler KC, Loquai C, Mohr P, Gutzmer R, Dummer R, Gabriel S, Wu CJ, Schadendorf D, Garraway LA. (2015) Genomic correlates of response to CTLA-4 blockade in metastatic melanoma. Science, 350(6257): 207–211. https://doi.org/10.1126/science.aad0095]

40 samples

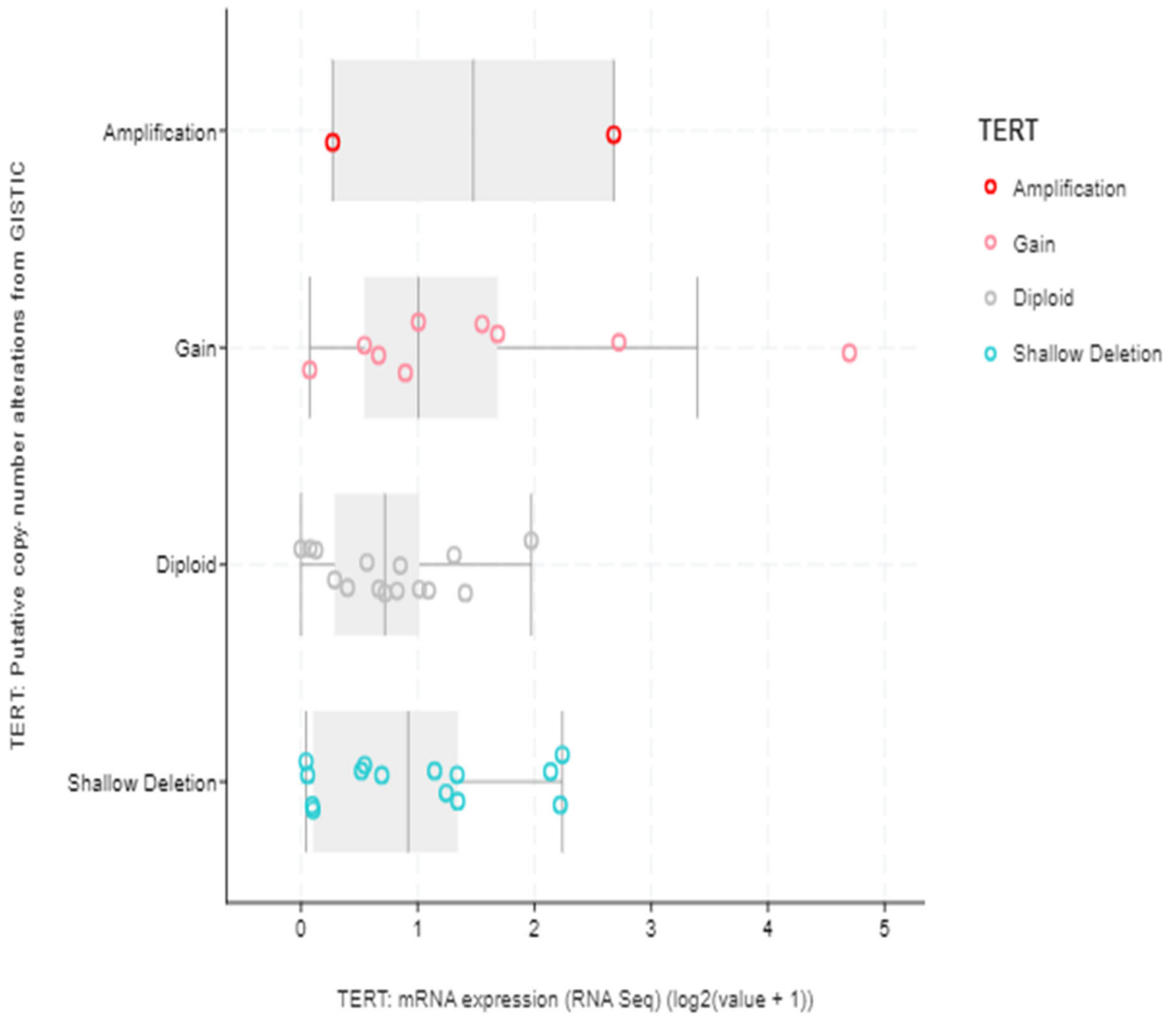

TCGA – HEPATOCELLULAR CARCINOMA [Hoadley KA, Yau C, Hinoue T, Wolf DM, Lazar AJ, Drill E, Shen R, Taylor AM, Cherniack AD, Thorsson V, Akbani R, Bowlby R, Wong CK, Wiznerowicz M, Sanchez-Vega F, Robertson AG, Schneider BG, Lawrence MS, Noushmehr H, Malta TM; Cancer Genome Atlas Network; Stuart JM, Benz CC, Laird PW. (2018) Cell-of-Origin Patterns Dominate the Molecular Classification of 10,000 Tumors from 33 Types of Cancer. Cell, 173(2): 291–304.e6. https://doi.org/10.1016/j.cell.2018.03.022]

348 samples

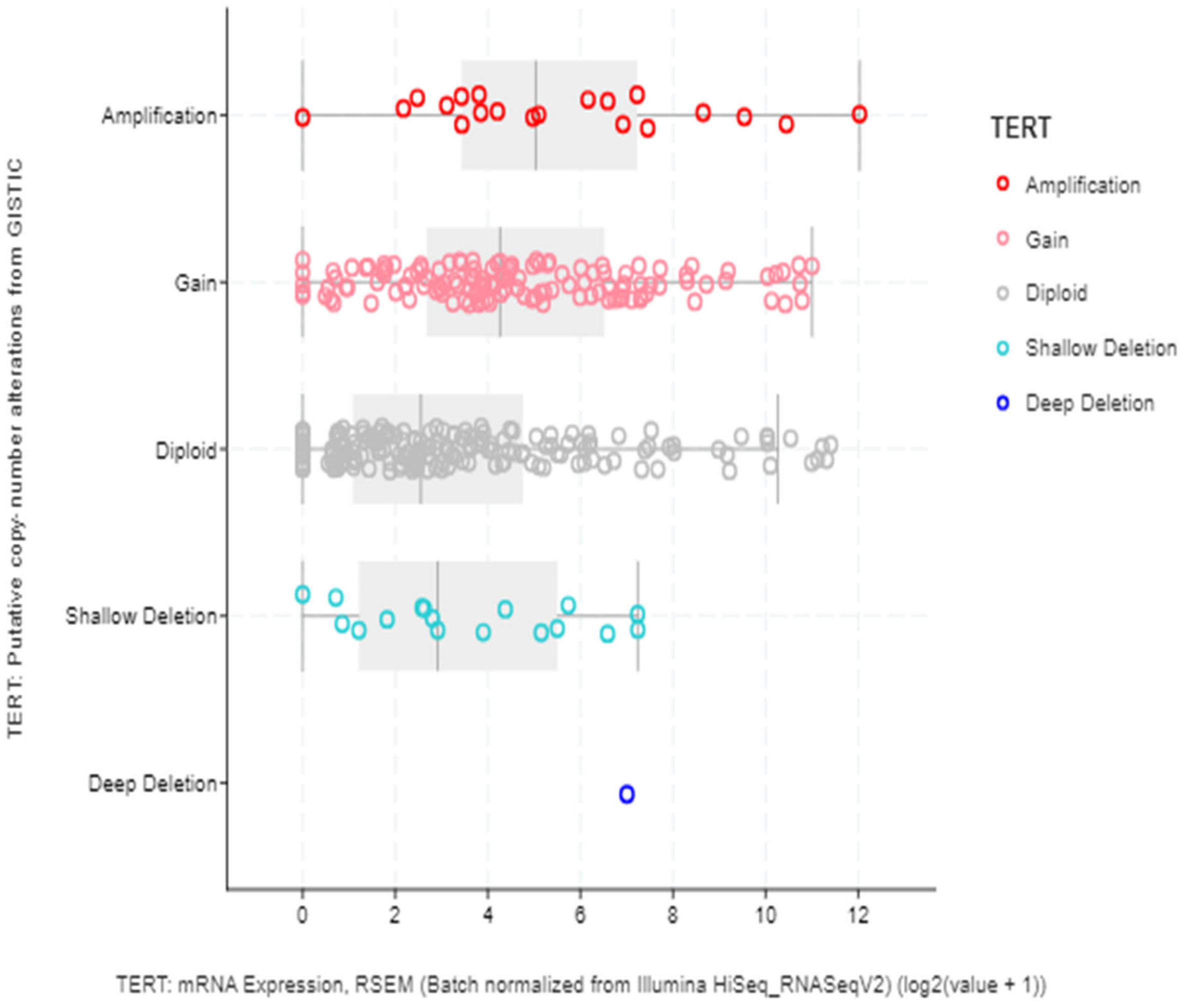

TERT mRNA expression has also been found to be significantly correlated with *TERT* amplification in Merkel cell carcinomas [*Xie H et al., TERT promoter mutations and gene amplification: promoting TERT expression in Merkel cell carcinoma. Oncotarget, 2014, 5: 10048-57. http://doi.org/10.18632/oncotarget.2491*], cervical carcinomas [*Zhang A et al., Amplification of the telomerase reverse transcriptase (hTERT) gene in cervical carcinomas. Genes Chromosomes Cancer, 2002, 34(3): 269-75. http://doi.org/10.1002/gcc.10071*], central nervous system embryonal tumors [*Fan X et al. hTERT gene amplification and increased mRNA expression in central nervous system embryonal tumors. Am J Pathol, 2003, 162(6): 1763-9. http://doi.org/10.1016/S0002-9440(10)64311-8*], and other tumors.
