# Supplementary material for: TERT Amplification a Risk Stratification Marker in Papillary Thyroid Carcinoma, Significantly Correlated with Tumor Recurrence and Survival
Source: Endocr Pathol. 2025 Apr 24;36(1):15. doi: 10.1007/s12022-025-09853-4 (PMC12021720; doi:10.1007/s12022-025-09853-4)
Supplement: Supplementary file 8 — Supplementary file6 (PDF 164 KB) [file 12022_2025_9853_MOESM6_ESM.pdf]

"Online resource 4" paper *Endocrine Pathology* DOI: 10.1007/s12022-025-09853-4

Impact of *BRAF* mutations in recurrence-free survival and risk of death of disease in PTC patients

Our results, in agreement with what we and others have previously reported [1,2-10], also suggest that *BRAF* mutations "*per se*" are insufficient to induce an aggressive tumor behaviour in PTC patients. Do not increase the risk of tumor recurrence or tumor related-death. The reported high prevalence of *BRAF* mutations in micro-PTC (20% to 52%) [7,8,10,11], which usually represent incidental findings and are considered to have the best prognosis of all thyroid carcinomas, seems to argue against the usefulness of *BRAF* detection in PTC risk stratification. Even studies of micro-PTC exhibiting aggressive behaviour, with an unusually high prevalence of extrathyroidal extension, LNMs, and advanced clinical stage, failed to reveal a correlation between *BRAF* mutation and tumor aggressiveness [11]. The fact that *BRAF* mutations are also present in many early-stage sporadic adult PTC with no evidence of dedifferentiation, which are cured by standard management protocols, showing a life expectancy similar to that of a normal population of similar age, provides as well further support for the notion that *BRAF* "alone" is insufficient to confer a fully aggressive phenotype. In neuroblastoma patients has been demonstrated that when tumors harbour telomere maintenance mechanisms in combination with other genetic events such as *RAS* and/or *p53* pathway mutations, the survival rates are much lower [12]. In our study, Kaplan-Meier analyses showed that when *BRAF* mutations co-occurred with *TPM* there was only a trend towards an association with an increased risk of tumor recurrence and lower survival, but when coexisted with *TERT* amplification or coexisted with mutations in any of the other oncogenic drivers investigated (*RAS* and/or *PIK3CA* and/or *TERTp*) and/or TERT amplification, there was a statistically significant association with a lower probability of recurrence-free survival and a greater risk of death of disease. Curiously, when the Kaplan-Meier analysis involved only PTCs without DMs the weak correlation found between *BRAF* + other events (*RAS* and/or *PIK3CA* and/or *TERTp and /or TERT* amplification) and a lower probability of recurrence-free survival or a greater risk of death of disease increased significantly [*BRAF* + other events (*RAS* and/or *PIK3CA* and/or *TPM and /or TERT* amplification)$\rightarrow$ recurrence Log-rank *P*=0.0043, and survival Log-rank *P*=0.0066].

Impact of *BRAF* mutational pattern on disease-related recurrence and survival in papillary thyroid carcinoma

Kaplan-Meier estimates analysis - *P* values

Genetic event	Recurrence	Survival
BRAF	0.0837	0.7812
BRAF* + *TPM	0.0871	0.0752
***BRAF* + *TERT* AMP**	**0.0208**	**0.0003**
***BRAF* + other genetic events**<br>(*RAS* and/or *PIK3CA* and/or *TPM* and/or *TERT* AMP)	**0.0497**	**0.0487**

Abbreviations: *TPM* , *TERT* promoter mutation; *TERT* AMP, *TERT* amplification.

Only statistically significant correlations or trends for an association have been included in the table. *P* values ≤ 0.05 (boldface) were considered statistically significant. *P* values between 0.05 and 0.18 were considered as a trend of correlation

Univariate analysis corroborated that *BRAF* mutations do not represent a statistically significant poor prognostic marker in our series of PTCs. *BRAF* mutations were not able to predict tumor-related death, and only showed a trend for an association with an increased risk of tumor relapse. However, when *BRAF* mutations coexisted with *TERT* amplification or with *TERT* amplification and *TPM* it did increase significantly the likelihood of tumor-related recurrence and death.

Univariate analysis - relative risk and likelihood ratio of disease-related recurrrencce or death associated with altered *BRAF* genotype

Cox proportional hazards model and Likelihood ratio test

Tumor recurrence

Genotype variables	RR/HR	95% CI	*P* value	Likelihood ratio	*P* value
BRAF	2.250	0.877-5.774	0.0914	2.973	0.0846
BRAF + OTHER (TPM and/or *RAS* and/or *PI3K)*	2.182	0.872-5.458	0.0951	2.618	0.1056
TERT AMP + BRAF	**3.223**	**1.140-9.109**	**0.0272**	**4.009**	**0.0452**
TPM + BRAF	2.182	0.872-5.458	0.0951	2.618	0.1056
TERT AMP + TPM + BRAF	**3.162**	**1.034-9.672**	**0.0434**	**3.253**	**0.0712**

Survival

Genotype variables	RR/HR	95% CI	*P* value	Likelihood ratio	*P* value
BRAF	1.164	0.381-3.554	0.7891	0.072	0.7878
BRAF + OTHER (TPM and/or *RAS* and/or *PI3K)*	2.345	0.806-6.820	0.1176	2.471	0.1158
TERT AMP + BRAF	**6.145**	**1.975-19.119**	**0.0017**	**8.734**	**0.0031**
TPM + BRAF	2.525	0.875-7.279	0.0863	2.841	0.0918
TERT AMP + TPM + BRAF	**3.162**	**1.034-9.672**	**0.0434**	**3.253**	**0.0712**

Abbreviations: RR/HR, Relative Risk / Hazard Ratio; 95% CI, 95% Confidence Interval; *TPM* , *TERT* promoter mutation; *TERT* AMP, *TERT* amplification.

P values ≤ 0.05 (boldface) were considered statistically significant. *P* values between 0.05 and 0.12 were considered as a trend of correlation

Multivariate analyses revealed that *TERT* amplification predicted tumor relapse and poor survival independently of *BRAF* mutations (**see table-5** main text).

REFERENCES:

1. Costa AM, Herrero A, Fresno MF, Heymann J, Alvarez JA, Cameselle-Teijeiro J, García-Rostán G. (2008) BRAF mutation associated with other genetic events identifies a subset of aggressive papillary thyroid carcinoma. Clin Endocrinol (Oxf), 68:618-634 DOI: 10.1111/j.1365-2265.2007.03077.x
2. Yan C, Huang M, Li X, Wang T, Ling R. (2019) Relationship between BRAF V600E and clinical features in papillary thyroid carcinoma. Endocr Connect, 8(7):988-996. DOI: 10.1530/EC-19-0246
3. Henke LE, Pfeifer JD, Ma C, Perkins SM, DeWees T, El-Mofty S, Moley JF, Nussenbaum B, Haughey BH, Baranski TJ, Schwarz JK, Grigsby PW. (2015) BRAF mutation is not predictive of long-term outcome in papillary thyroid carcinoma. Cancer Med, 4(6):791-799. DOI: 10.1002/cam4.417
4. Czarniecka A, Kowal M, Rusinek D, Krajewska J, Jarzab M, Stobiecka E, Chmielik E, Zembala-Nozynska E, Poltorak S, Sacher A, Maciejewski A, Zebracka-Gala J, Lange D, Oczko-Wojciechowska M, Handkiewicz-Junak D, Jarzab B. (2015) The Risk of Relapse in Papillary Thyroid Cancer (PTC) in the Context of BRAFV600E Mutation Status and Other Prognostic Factors. PLoS One, 10(7):e0132821 DOI: 10.1371/journal.pone.0132821
5. Sapio,MR, Posca D, Troncone G, Pettinato G, Palombini L, Rossi G, Fenzi G, Vitale M. (2006) Detection of BRAF mutation in thyroid papillary carcinomas by mutant allele-specific PCR amplification (MASA). Eur J Endocrinol, 154:341-348. DOI: 10.1530/eje.1.02072
6. Puxeddu E, Moretti S, Elisei R, Romei C, Pascucci R, Martinelli M, Marino C, Avenia N, Rossi ED, Fadda G, Cavaliere A, Ribacchi R, Falorni A, Pontecorvi A, Pacini F, Pinchera A, Santeusanio F. (2004) $BRAF^{V599E}$ mutation is the leading genetic event in adult sporadic papillary thyroid carcinomas. J Clin Endocrinol Metab, 89:2414-2420. DOI: 10.1210/jc.2003-031425
7. Liu RT, Chen YJ, Chou FF, Li CL, Wu WL, Tsai PC, Huang CC, Cheng JT. (2005) No correlation between $BRAF^{V600E}$ mutation and clinicopathological features of papillary thyroid carcinomas in Taiwan. Clin Endocrinol, 63:461-466. DOI: 10.1111/j.1365-2265.2005.02367.x
8. Trovisco V, Soares P, Preto A, Vieira de Castro I, Lima J, Castro P, Máximo V, Botelho T, Moreira S, Meireles AM, Magalhães J, Abrosimov A, Cameselle-Teijeiro J, Sobrinho-Simões M. (2005) Type and

prevalence of BRAF mutations are closely associated with papillary thyroid carcinoma histotype and patients age but not with tumour aggressiveness. Virchows Archive, 446:589-595. DOI: 10.1007/s00428-005-1236-0

9. Fugazzola L, Puxeddu E, Avenia N, Romei C, Cirello V, Cavaliere A, Faviana P, Mannavola D, Moretti S, Rossi S, Sculli M, Bottici V, Beck-Peccoz P, Pacini F, Pinchera A, Santeusanio F, Elisei R. (2006) Correlation between B-RAFV600E mutation and clinico-pathologic parameters in papillary thyroid carcinoma: data from a multicentric Italian study and review of the literature. Endocr Relat Cancer, 13:455-464. DOI: 10.1677/erc.1.01086

10. Sedliarou I, Saenko V, Lantsov D, Rogounovitch T, Namba H, Abrosimov A, Lushnikov E, Kumagai A, Nakashima M, Meirmanov S, Mine M, Hayashi T, Yamashita S. (2004) The BRAFT1796A transversion is a prevalent mutational event in human thyroid microcarcinoma. Int J Oncol, 25:1729-1735 DOI: 10.3892/ijo.25.6.1729

11. Kim TY, Kim WB, Son, JY, Rhee YS, Gong G, Cho YM, Kim SY, Kim SC, Hong SJ, Shong YK. (2005) The BRAFV600E mutation is not associated with poor prognostic factors in Korean patients with conventional papillary microcarcinoma. Clin Endocrinol, 63:588-593 DOI: 10.1111/j.1365-2265.2005.02389.x

12. Ackermann S, Cartolano M, Hero B, Welte A, Kahlert Y, Roderwieser A, Bartenhagen C, Walter E, Gecht J, Kerschke L, Volland R, Menon R, Heuckmann JM, Gartlgruber M, Hartlieb S, Henrich KO, Okonechnikov K, Altmüller J, Nürnberg P, Lefever S, de Wilde B, Sand F, Ikram F, Rosswog C, Fischer J, Theissen J, Hertwig F, Singhi AD, Simon T, Vogel W, Perner S, Krug B, Schmidt M, Rahmann S, Achter V, Lang U, Vokuhl C, Ortmann M, Büttner R, Eggert A, Speleman F, O'Sullivan RJ, Thomas RK, Berthold F, Vandesompele J, Schramm A, Westermann F, Schulte JH, Peifer M, Fischer M. (2018) A mechanistic classification of clinical phenotypes in neuroblastoma. Science, 362(6419):1165-1170. DOI: 10.1126/science.aat6768
